# Analysis of Genetic Diversity in Adzuki Beans (*Vigna angularis*): Insights into Environmental Adaptation and Early Breeding Strategies for Yield Improvement

**DOI:** 10.3390/plants12244154

**Published:** 2023-12-13

**Authors:** Xiaohan Wang, Yu-Mi Choi, Young-ah Jeon, JungYoon Yi, Myoung-Jae Shin, Kebede Taye Desta, Hyemyeong Yoon

**Affiliations:** National Agrobiodiversity Center, National Institute of Agricultural Sciences, Rural Development Administration, Jeonju 54874, Republic of Korea; wangxiaohan0530@gmail.com (X.W.); ymchoi@rda.go.kr (Y.-M.C.); yjeon@korea.kr (Y.-a.J.); naaeskr@korea.kr (J.Y.); smj1204@korea.kr (M.-J.S.);

**Keywords:** adzuki bean, number of seeds per pod, number of pods per plant, 100-seed weight, domestication selection, crop evolution, phylogeography

## Abstract

Adzuki beans are widely cultivated in East Asia and are one of the earliest domesticated crops. In order to gain a deeper understanding of the genetic diversity and domestication history of adzuki beans, we conducted Genotyping by Sequencing (GBS) analysis on 366 landraces originating from Korea, China, and Japan, resulting in 6586 single-nucleotide polymorphisms (SNPs). Population structure analysis divided these 366 landraces into three subpopulations. These three subpopulations exhibited distinctive distributions, suggesting that they underwent extended domestication processes in their respective regions of origin. Phenotypic variance analysis of the three subpopulations indicated that the Korean-domesticated subpopulation exhibited significantly higher 100-seed weights, the Japanese-domesticated subpopulation showed significantly higher numbers of grains per pod, and the Chinese-domesticated subpopulation displayed significantly higher numbers of pods per plant. We speculate that these differences in yield-related traits may be attributed to varying emphases placed by early breeders in these regions on the selection of traits related to yield. A large number of genes related to biotic/abiotic stress resistance and defense were found in most quantitative trait locus (QTL) for yield-related traits using genome-wide association studies (GWAS). Genomic sliding window analysis of Tajima’s D and a genetic differentiation coefficient (Fst) revealed distinct domestication selection signatures and genotype variations on these QTLs within each subpopulation. These findings indicate that each subpopulation would have been subjected to varied biotic/abiotic stress events in different origins, of which these stress events have caused balancing selection differences in the QTL of each subpopulation. In these balancing selections, plants tend to select genotypes with strong resistance under biotic/abiotic stress, but reduce the frequency of high-yield genotypes to varying degrees. These biotic/abiotic stressors impact crop yield and may even lead to selection purging, resulting in the loss of several high-yielding genotypes among landraces. However, this also fuels the flow of crop germplasms. Overall, balancing selection appears to have a more significant impact on the three yield-related traits compared to breeder-driven domestication selection. These findings are crucial for understanding the impact of domestication selection history on landraces and yield-related traits, aiding in the improvement of adzuki bean varieties.

## 1. Introduction

Adzuki bean (*Vigna angularis* L.) (2n = 22) is a self-pollinating annual legume crop [1] widely cultivated in Eastern Asia. Archaeological evidence indicate that the earliest remains of adzuki beans are from the Neolithic site in Liangchengzhen, Shandong, China [2]. Domestication of the bean has been suggested to have taken place in China about 12,000 years ago [3]. Adzuki beans are mainly cultivated and consumed in China, Japan, and Korea and has been introduced to other continents in recent decades. Adzuki beans are now distributed in more than 30 countries, with a planting area of nearly 700,000 hectares [1,4]. Adzuki beans are highly adaptable to the environment and are drought tolerant, but not flood tolerant. Therefore, in the early days, small areas were often planted, which included barren lands, saline-alkaline lands, dry lands, mountains, and roadsides, while crops with poor adaptability were planted in fields where the water and fertilizer inputs could be more easily controlled.

Adzuki beans are rich in nutrients, sweet in taste, and are used to make traditional pastries due to their unique flavor [5]. Due to its low calorie and fat content, it is increasingly valued in modern times for the development of new pastries. In addition, adzuki beans are rich in protein, vitamins, flavonoids, and minerals. They have been used in medicine since ancient times to treat inflammation, antioxidant, immune regulation, and blood sugar and blood lipid regulation [6,7,8,9,10]. Wild adzuki beans and cultivated adzuki beans are mainly self-pollinating, and the occurrence rate of outcrossing is 1% [11].

Adzuki bean breeding have mainly focused on yield, seed coat color, and the flowering period [11]. The seed weight, number of seeds per pod and number of pods per plant of legume crops are three important component traits that directly determine yield [12]. The three yield components of adzuki bean exhibit a high positive correlation with the total yield, while there is a small negative correlation among the three yield-related traits [11]. Early breeders used to select large and plump seeds for subsequent sowing [13,14]. Therefore, 100-seed weight can be considered as the most domesticated trait. In plants, grain weight regulatory pathways have been extensively studied. The seed size or 100-seed weight has been shown to be jointly regulated via these 12 pathways [15,16,17]. Among them, the ubiquitin–proteasome pathway mediates integration development, and the transcription factor (TF) pathway, the guanine nucleotide-binding proteins (G proteins) pathway, and the Gibberellic acid (GA) pathway jointly control cell division. The auxin (IAA) pathway promotes seed development by inducing cell enlargement. The mitogen-activated protein kinase (MAPK) pathway, the brassinosteroid (BR) pathway, the abscisic acid (ABA) pathway, and the HAIKU (IKU) pathway jointly regulate endosperm and seed development. The jasmonate (JA) pathway and the cytokinins (CKs) pathway on the other hand inhibit seed development. More than 80 genes in 12 pathways have been shown to be involved in regulating the development of seeds. The number of ovules which develop into mature seeds after fertilization is determined via the ovule differentiation process. Ovule fertility, pollen/pollen tube development, and interaction determine the fertilization rate of the ovule. The process of ovule development into seeds after fertilization is affected by the physiological conditions of the plant and the external environment [18]. The number of pods per plant in legumes is largely regulated by the number of pods in a single node [19,20,21,22]. From flowering to the early stages of ovule formation, exogenous cytokinin treatment has been shown to significantly promote the number of pods per segment [23,24]. These studies point to a critical role for cytokinins in legume pod development.

A large number of studies have used an amplified fragment length polymorphism (AFLP), simple sequence repeats (SSR), and other molecular markers to analyze the genetic diversity of adzuki bean [25,26,27,28,29,30]. These studies indicate that East Asia is the center of genetic diversity in cultivated adzuki beans. Adzuki beans from China, Japan, and South Korea have the highest diversity, and there is a high level of genetic differentiation among the adzuki bean germplasms in these three regions, indicating that the adzuki bean germplasms in the three regions have undergone long-term independent domestication. Adzuki beans have been domesticated via long-term selection in terms of seed dormancy, pod dehiscence, gigantism, plant type, earliness, yield potential, and pigmentation. This is the result of both adapting to the planting environment as well as to human preferences. The genetic diversity of wild adzuki bean germplasm is higher than that of cultivated species, indicating that the use of wild species still has the potential to improve existing cultivated species. A domestication bottleneck was observed in adzuki bean, but 86% of wild species diversity was reported to be retained [30].

There are hitherto unresolved issues in the study of genetic diversity in adzuki beans. For example, whole genome sequencing data has not been well applied to the genetic diversity analyses of adzuki bean. A large number of studies have reported differences in yield-related traits among adzuki bean germplasms that have been independently domesticated in various domestication centers, but the exact cause of this difference has not yet been found. We collected 366 adzuki bean local varieties from three countries, China, Japan and South Korea, and used GBS data to analyze the specific reasons for the differences in yield traits in adzuki bean domesticated at different regions.

## 2. Results

### 2.1. Genotyping by Sequencing

We performed GBS on 366 adzuki bean accessions collected in Korea, China, and Japan. A total of 9808 SNPs were obtained via variant calling and quality control, and after filtering, 6586 SNPs located on 11 chromosomes in the genome were finally obtained. The Vigan1.1 genome available at NCBI is 448.36 Mb in size. Calculations based on the average Linkage disequilibrium (LD) decay distance (679,517 bp) indicated that the coverage of the entire genome by 6586 SNPs was 9.98 times (Figure S1).

### 2.2. Population Structure

**Figure 1:**
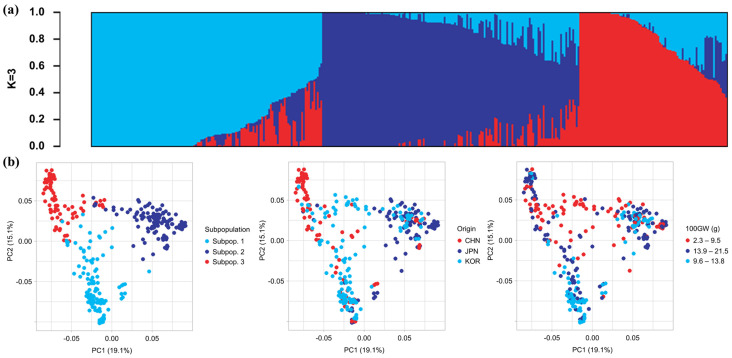
The genetic characteristics of 366 adzuki bean landraces were significantly correlated with country of origin and grain weight. (**a**) Population structure analysis of 366 adzuki bean landraces resulted in three subpopulations. (**b**) Principal component analysis (PCA) plot of genetic characteristics of 366 adzuki bean germplasms. In the three PCA plots, each subpopulation, each origin, and each grade of 100-grain weight are colored, respectively. There is a significant correlation between origin, 100-seed weight, and genetic characteristics.

We used genetic data to analyze the population structure for k = 2 to 10. The structure harvester results showed that when the number of subpopulations (K) in the population is 3, Delta K was the highest, which was 5.974 (Figure 1). After coloring both the differences in origin and 11 agronomic traits, it was found that the origin and 100-seed weight were differentially distributed among different subpopulations. We demonstrated these correlations using chi-square tests. Comparing the distribution of subpopulations in various origins, it was found that Chinese germplasm has the widest distribution range, with most germplasms concentrated in subpopulation 3, and a small number of germplasms were widely distributed in various regions. Korean germplasm had the most even distribution and was distributed in all three subpopulations, albeit a majority of them were distributed in subpopulation 1. Although Japan retains individual germplasms of subpopulation 1 and subpopulation 3, most of them were distributed in subpopulation 2. Referring to the geographical distribution of the three landrace subpopulations, we believe that subpopulation 1 was mainly selected for long-term domestication in Korea, subpopulation 2 was domesticated in Japan, and subpopulation 3 was domesticated in China.

### 2.3. Genetic and Phenotypic Diversity

We performed genetic diversity analyses of germplasm populations from each origin. The results showed that Chinese germplasm had the highest genetic diversity, number of different alleles (1.983), number of effective alleles (1.477), Shannon’s information index (0.447), observed heterozygosity (0.007), and expected heterozygosity (0.290). This was followed by the Korean germplasm, with the number of different alleles (1.972), number of effective alleles (1.410), Shannon’s information index (0.407), and expected heterozygosity (0.259), while the observed heterozygosity (0.004) was the lowest among the three origins. Japanese germplasm had the lowest genetic diversity, with the number of different alleles (1.925), number of effective alleles (1.353), Shannon’s information index (0.349), and expected heterozygosity (0.221) all being the lowest, while the observed heterozygosity (0.004) was ranked second. Adzuki bean resources indicated Ho to be much smaller than He and that the loss of heterozygosity was caused by high inbreeding or self-pollination.

Genetic diversity analysis was also performed for each subpopulation and the results showed that subpopulation 2, domesticated in Japan, had the highest genetic diversity (Table S1; Figure S2). The number of different alleles (1.998), number of effective alleles (1.466), Shannon’s information index (0.455), observed heterozygosity (0.006), and expected heterozygosity (0.292) are all the highest. Followed by the Chinese-domesticated subpopulation 3, the number of effective alleles (1.4), Shannon’s information index (0.396), and expected heterozygosity (0.251) are all second, while the number of different alleles (1.959) and observed heterozygosity (0.004) is the lowest. The Korean-domesticated subpopulation 3 has the lowest genetic diversity, number of effective alleles (1.39), Shannon’s information index (0.391), and expected heterozygosity (0.246). The number of different alleles (1.988) and observed heterozygosity (0.005) are second.

We compared the quantitative trait differences between the three subpopulations using the SNPs’ data (Table 1). The quantitative traits with significant differences among the three subpopulations included the number of pods per plant, number of seeds per pod, 100-seed weight, yield per plant, flowering date, and maturity date. Subpopulation 3 had the highest number of pods per plant, with an average of 42.345, and the earliest flowering period, with 60 days. Subpopulation 2 had the highest number of seeds per pod, with an average of 7.178, and the earliest harvest period, with 106.838 days. Subpopulation 1 had the highest 100-seed weight, with an average of 14.187 g, and the highest harvest yield per plant, with an average of 36.375 g. The significant differences in the number of seeds per pod (NOSPP), number of pods per plant (NOPPP), and 100-seed weight among the three subpopulations could have been due to the early breeders in China, Japan, and Korea focusing on domesticating and focusing on different traits.

Compared to the normal distribution, the skewness of the number of pods per plant and the harvest amount per plant is much greater than zero, indicating a right-skewed distribution, while the negative skewness of the maturity date (days after sowing, DAS) means a left-skewed distribution. The kurtosis value of the two traits is greater than zero, indicating that the sample is more concentrated near the peak. The skewness and kurtosis of other traits are close to zero, indicating that the distribution is close to a normal distribution.

### 2.4. Gene Flow Analysis and Phylogenetic Analysis

Phylogenetic analysis was conducted on 366 local varieties from China, Japan, and Korea using the NJ model and three germplasms each of mung bean, cowpea, and ricebean were added as outgroups, used to analyze the evolutionary relationships between various species and subpopulations (Figure S3). The results showed that accession number 35 in CHN subpopulation 1 had the closest genetic distance to the differentiation node of the outgroup and was the oldest adzuki bean accession among these accessions. The evolutionary order among subpopulations is subpopulation 1, which was introduced to Korea from China and underwent secondary domestication in Korea, followed by subpopulation 3, domesticated in China, and finally, subpopulation 2, domesticated in Japan.

In the gene flow analysis, to understand the flow direction of each subpopulation in detail, the 366 accessions were divided into nine parts. Due to the small sample size in JPN3, any errors that occurred were removed during the calculation process. The direction of migration was determined based on the amount of migration in both directions. The results showed that the average migration rate from KOR3 to CHN1 was 111.4, which was less than the average migration rate in the reverse direction (152.5); the average migration rate from CHN3 to CHN2 was 96.8, which was greater than the average migration rate in the reverse direction (49.3); the average migration rate from JPN2 to CHN3 was 98.1, which was greater than the average migration rate in the reverse direction (69.3); the average migration rate from CHN2 to JPN1 was 119.4, which was greater than the average migration rate in the reverse direction (67.3); the average migration rate from CHN2 to JPN2 was 105.7, which was greater than the average migration rate in the reverse direction (77.4); the average migration rate of KOR1 was 131.1, which was greater than the average migration rate of the reverse direction (77.1); the average migration rate of JPN1 to KOR2 was 130.9, which was greater than the average migration rate of the reverse direction (45.9); and the average migration rate of JPN2 to CHN3 was 173.6, which was greater than the average migration rate of the reverse direction. The average migration rate was 104.8 (Table S1).

### 2.5. GWAS

**Figure 2:**
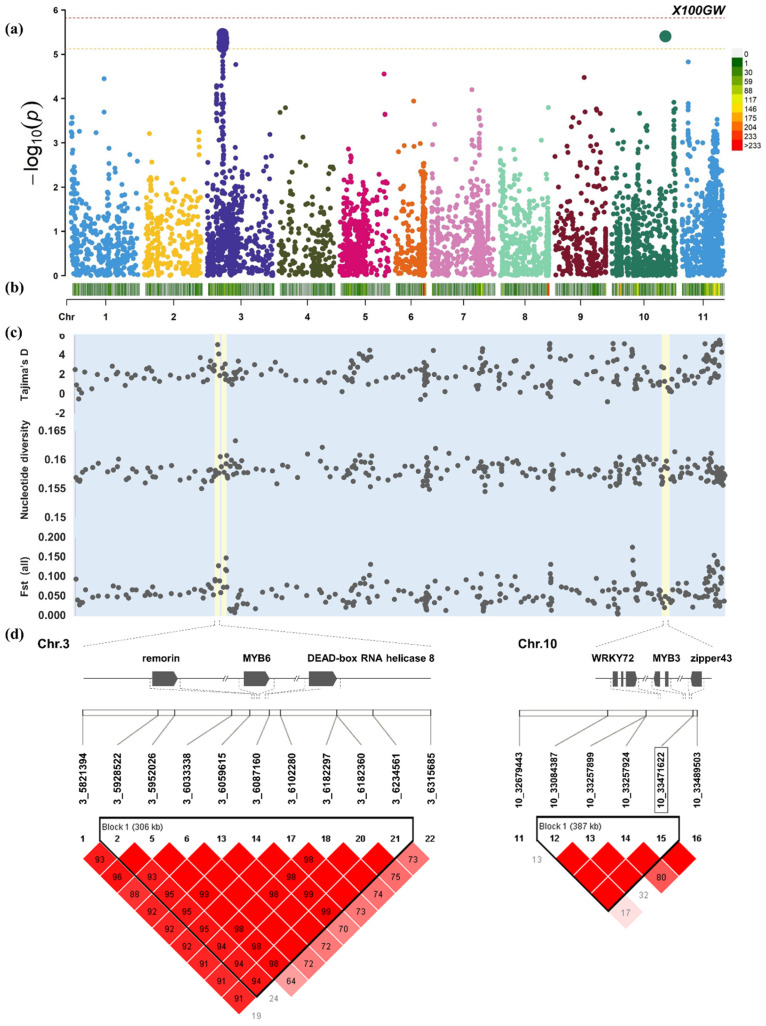
The 100GW candidate genes and genetic diversity in the QTL regions. (**a**) GWAS results of 366 adzuki bean 100GW. (**b**) Distribution of 6586 SNPs in the genome. (**c**) Genomic sliding window analysis of Tajima’s D, nucleotide diversity, and genetic differentiation index (Fst) of 366 adzuki bean accessions. QTL located on chromosomes 3 and 10 are marked in light yellow. (**d**) Determine the screening range of 100GW candidate genes based on haplotype analysis.

We used the mixed linear model (MLM) model to conduct GWAS (Figure 2). The heritability of 100-seed weight was 82.2%. A QTL for 100-seed weight was found on chromosome 3 (Chr.3: 9,757,052–10,654,699). There were three concatenated haploid blocks in this QTL with SNPs exceeding the Bonferroni corrected threshold. In the first haploid block (117 kb), there are three proteins, a late up-regulated protein (LURP) called LURP-one-related 15-like, a protein kinase 2A, and a chloroplastic-like and uncharacterized protein. The size of the second haploid block was 207 kb. Within its scope are a protein LURP-one-related 15-like, a signal-transducing adapter molecule 2-like, a regulatory-associated protein of target of rapamycin TOR 1-like, and an alanine–glyoxylate aminotransferase 2 homolog 3, mitochondrial-like. The size of the third haploid block was 106 kb, containing one protein LURP-one-related 15-like, two uncharacterized proteins, and one protein NSP-INTERACTING KINASE 1-like.

In the 100-seed weight QTL located on chromosome 10 (Chr.10: 32,679,443–33,489,503), the haploid block size is 387 kb. Candidate genes that may be related to 100-seed weight include probable heptapeptide-conserved sequence WRKYGOK (WRKY) transcription factor 72, transcription factor v-Myb myeloblastosis viral oncogene homolog (MYB) MYB3-like, and basic leucine zipper 43-like. Among them, basic leucine zipper 43-like is closest to sentinel SNP.

Among the 100-seed weight QTL on chromosome 11, sentinel SNP is located in a 176 kb haploid block. Candidate genes located on the haploid include hexokinase-2-like, casparian strip domain protein (CASP) CASP-like protein 4B1, two uncharacterized proteins, and four tandem soyasapogenol B glucuronide galactosyltransferase-like.

**Figure 3:**
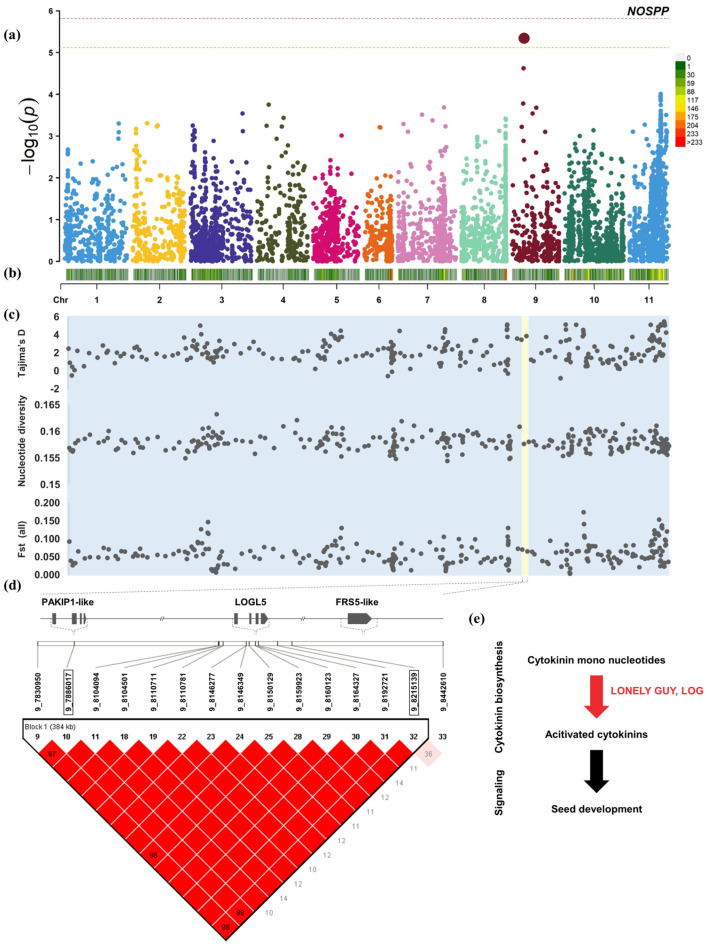
NOSPP candidate genes and genetic diversity in the QTL region. (**a**) GWAS results of 366 adzuki bean NOSPP. (**b**) Distribution of 6586 SNPs in the genome. (**c**) Genomic sliding window analysis of Tajima’s D, nucleotide diversity, and genetic differentiation index (Fst) of 366 adzuki bean accessions. QTL located on chromosome 9 is marked in light yellow. (**d**) Determine the screening of NOSPP candidate genes based on haplotype analysis. (**e**) The mechanism of candidate genes most likely to affect NOSPP based on gene function screening. Physiological processes mediated by candidate genes are marked in red.

The heritability of the number of seeds per pod was only 35.3%. A QTL for the number of seeds per pod was mapped on chromosome 9: 7,830,950–8,442,610 (Figure 3). In the 384 kb interval, two non-adjacent SNPs exceeded the threshold. The SNP located on chromosome 9: 7,886,017 has a *p* value that exceeds the threshold and is flanked by a p21-activated protein kinase-interacting protein 1-like (PAKIP1-like), which is related to the regulation of cell signaling pathways. Furthermore, another over-threshold SNP (Chr.9: 8,215,139) was found flanking the cytokinin-related probable cytokinin riboside 5′-monophosphate phosphoribohydrolase LOGL5 and protein Fatty Acyl-CoA Reductase 1-related sequence 5-like.

**Figure 4:**
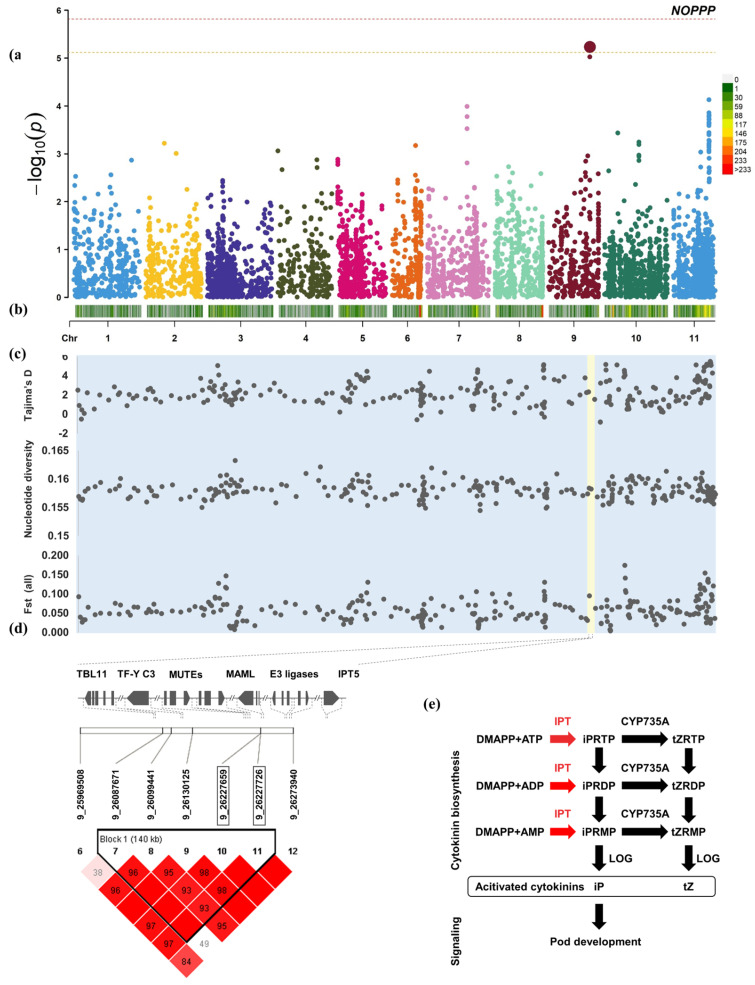
NOPPP candidate genes and genetic diversity in the QTL region. (**a**) GWAS results of 366 adzuki bean NOPPP. (**b**) Distribution of 6586 SNPs in the genome. (**c**) Genomic sliding window analysis of Tajima’s D, nucleotide diversity, and genetic differentiation index (Fst) of 366 adzuki bean accessions. QTL located on chromosome 9 is marked in light yellow. (**d**) Determine the screening range of NOPPP candidate genes based on haplotype analysis. (**e**) The mechanism of candidate genes most likely to affect NOPPP based on gene function screening. Physiological processes mediated by candidate genes are marked in red. DMAPP: dimethylallyl pyrophosphate; IPT, isopentenyltransferases; iPRTP: isopentenyladenine riboside 5-triphosphate; iPRDP: isopentenyladenine riboside 5-diphosphate; iPRMP: isopentenyladenine riboside 5-monophosphate; CYP735A, cytochrome P450 monooxygenase; LOG: LONELY GUY; tZRTP, trans-zeatin riboside 5-triphosphate; tZRDP, trans-zeatin riboside 5-diphosphate; tZRMP, trans-zeatin riboside 5-monophosphate; iP, N6-(Δ2-isopentenyl)adenine; tZ: trans-zeatin.

The heritability of the pod number per plant was 42.2%. According to the results of GWAS and haploid analysis of the number of pods per plant, seven candidate genes that may be related to the number of pods per plant were found on chromosome 9 from 25,969,508 to 26,273,940 (Figure 4). They are the trichome birefringence-like 11 protein (TBL11), nuclear transcription factor Y subunit C-3-like, two transcription factors MUTE-like, neurogenic protein mastermind-like (MAMLs), and two probable E3 ubiquitin-protein ligase.

## 3. Discussion

### 3.1. Migration History and Genetic Diversity Status of Adzuki Bean

Adzuki bean are common knowledge of crop migration influenced by economic and cultural exchanges. After adzuki bean cultivation became popular in various regions in the early days, the populations maintained a continuous flow relationship. This process was accompanied via continuous domestication selection by breeders and farmers. Gene flow analysis only applies to pairwise flow relationships between populations. We utilized rooted phylogenetic trees containing closely related outgroups to determine the phylogenetic relationships between the studied accessions. Based on the results of gene flow and phylogenetic analyses, we proposed the most likely migration model of adzuki bean. Accession number CHN1_35 was the oldest accession and was first domesticated (Figure S3). CHN1 domestication selected CHN3, which occupies most of the market in China (Table S2). The accession CHN2 differentiated from CHN3 was introduced to South Korea and Japan and was domesticated into the mainly cultivated KOR1 and JPN2 in South Korea and Japan, respectively, later being domesticated again in Japan. The excellent germplasm of JPN2 was once again introduced to China to improve CHN3. These events are reflected in the average migration rates derived from gene flow analysis. In gene flow analysis, CHN3 appears to be derived from JPN2. According to the results of gene flow analysis, JPN2 comes from CHN2. If CHN2 comes from CHN3, it is contradictory that CHN3 comes from JPN2. Therefore, according to the results of phylogenetic analysis, CHN3 actually appeared after domestication selection from CHN1. The contradictions in the gene flow results may be due to the fact that JP2 showed excellent traits and was reused to improve CHN3.

Subpopulation 2 shows the highest LD decay distance. This means that subpopulation 2 may have experienced more selection clearance and background selection. The LD decay distance of adzuki bean landrace populations collected near three domestication centers reached 680 kb. We compared the LD decay distance of adzuki bean with other self-pollinated crops. The LD decay distance of rice is approximately 100 kb to 200 kb [31,32,33]. The natural population decay distance of soybean is 200~375 kb [34,35]. The decay distance of the natural population of mung bean is 57.6 kb~100 kb [36]. Overall, the genetic diversity of adzuki bean populations was not high, which is consistent with the description of genetic diversity of adzuki bean cultivars and landraces in other studies [4].

### 3.2. Candidate Genes for Yield-Related Traits

In the GWAS results, WRKY transcription factor 72 on chromosome 10 may be related to the IKU pathway. The seed size of the *MINI3* gene mutant was significantly smaller than that of the control group [37]. The VQ motif of IKU pathway member IKU1 specifically binds to the C-terminal WRKY of WRKY superfamily group I WRKY proteins and the WRKY domain of group IIc WRKY proteins and significantly enhances the regulation of CYTOKININ OXIDASE 2 (CKX2), thereby enhancing the control of cytokinin homeostasis in the endosperm [38,39].

In addition, according to the BioGRID 4.4.224 (https://thebiogrid.org/, accessed on 25 August 2023) data, WRKY72 has an interactive relationship with MAPK6. In the Mitogen-Activated Protein Kinase (MAPK) Pathway, MAPK6, MAPK kinase (MAPKK), and MAPK kinase kinase (MAPKKK) form the MAPK cascade [40,41]. The MAPK cascade balances rice grain size via cell proliferation and BR signaling and participates in the signaling pathway control of the grain shape.

MYB3 such as MYB56 and MYB37 belong to R2R3 MYB. Both MYB56 and MYB37 have effects on seed size. Moreover, MYB56 regulates the expression of multiple genes involved in cell development, including pectinesterase, the pectinesterase inhibitor family, and various glycosyltransferases [42]. MYB37 is involved in the ABA signaling pathway and has also been reported to promote endosperm development by enhancing ABA sensitivity [43]. ABI5 (ABA-Insensitive 5) is also a basic leucine zipper transcription factor [44]. ABI5 acts as an integrator of signaling with other phytohormones, regulating auxin, cytokinin, and brassinosteroid signaling as well as metabolism at different developmental stages.

Among the QTL for the number of seeds per pod, p21-activated kinases 1 (PAK1) PAK1 P21 mediates cell proliferation, migration, and survival in animal cells and is an effective therapeutic target for cancer treatment. However, the specific function of this gene in plants has not been reported [45]. In plant cells, cytokinin riboside 5′-monophosphate phosphoribohydrolase (LOGL5) directly converts inactive cytokinin nucleotides into active free bases and is used to maintain cytokinin homeostasis [46]. Cytokinins regulate pistil and fruit development, inducing an increase in the number of pods and ovules [47,48]. Cytokinins and their negative regulators—LOGs—have a major impact on inflorescence structure and yield. Plants overexpressing LOGs showed reduced panicle size, abnormal branching, and defects in flower development. The loss of abundant members of the LOG family may lead to developmental defects, and the presence of additional abundant family members may improve the phenotype to a certain extent [49,50]. FAR1-related sequences (FRS) have been shown to play important roles in a variety of cellular processes. These include light signaling, circadian clock and flowering time regulation, shoot meristem and flower development, etc. [51]. FRS5-like may regulate the number of seeds per pod of adzuki bean by participating in the synthesis of strigolactone and cytokinins. Studies have confirmed that its homologous genes are involved in regulating branching and plant body structure and are specifically expressed in different pod development stages [52].

The *TBL* gene may promote the synthesis and deposition of secondary wall cellulose by affecting the esterification state of the polymer, pectin [53]. Pectin methyl esterification has been reported to affect pollen tube growth and pod elongation [54]. Abscisic acid plays an important role in regulating plant embryo morphogenesis and catalyzing seed maturation [55]. The nuclear transcription factor Y subunit C-3-like was found to be involved in the abscisic acid biosynthesis process and was significantly upregulated after fertilization [56]. The transcription factor MUTE encodes a basic helix-loop-helix (bHLH) protein, which switches cells to a differentiated state and is a precise differentiation regulator necessary for stomatal development [57]. In mammals, neurogenic protein mastermind-like proteins (MAMLs) act to activate late-differentiation muscle genes. MAMLs function in the Notch signaling pathway and are involved in cell fate determination, playing an important role in the regulation of embryonic development [58,59]. The C3HC4 conserved domain was found in both probable E3 ubiquitin-protein ligase gene sequences. The C3HC4-type RING finger E3 ligases regulate many cellular processes, including homeostasis, development, cell division, growth, hormonal responses, and stress responses, whereas the RING finger E3 ligase has been confirmed to regulate grain size in wheat, Arabidopsis, and peanut [60,61,62].

The number of pods per plant is controlled by the number of pods in a single node, which is related to the number of flowers in each node. During flowering, adenylate isopentenyltransferase 5 (IPT5) plays an important role in the biosynthesis of cytokinin, and its involvement in regulation has been widely confirmed [63].

### 3.3. Different Subpopulations Were Found to Have Different Traces of Domestication Selection on Yield-Related Traits in the Genome

Domesticated subpopulations in each region showed significant differences in three yield-related traits. In addition, candidate gene intervals in different subpopulations showed different Tajima’s D. This means that yield-related loci in different subpopulations are under domestication selection with different strengths. To further explore the factors influencing the evolutionary dynamics of these loci, we scanned the haploid blocks in which these loci reside. The aim is to find other genes that may have been selected via domestication to analyze the possibility that either yield-related loci were hitchhiking or background selection was involved during the selection process of these genes. Early breeders in various regions seemed to have different strategies for improving adzuki bean yields. The number of pods per plant of subpopulation 2, which was secondarily domesticated in Japan, was only 36.194, which was the lowest among the accessions from the three origins, while the number of seeds per pod was the highest, with an average of 7.178. The alleged strategy to improve adzuki bean yield or quality in Korea seems to be screening for larger seeds. The number of pods per plant (39.351) of subpopulation 1 that was secondarily domesticated in Korea was moderate. When the number of seeds per pod (6.678) was the lowest among the three origins, the average 100-seed weight of subpopulation 1 was, however, the highest, at 14.187 g. Early Chinese breeders may have focused their attention on developing germplasm based on the number of pods per plant. The characteristics of subpopulation 3, domesticated in China, included the number of pods per plant at the highest, with an average of 42.345; the number of seeds per pod (6.889) second, and the weight of 100 seeds being the lowest, at only 10.284 g. We calculated the yield per plant of each germplasm based on “yield per plant = weight of seeds/100 × Number of pods per plant × Number of seeds per pod”. The results showed that the Korean-domesticated subpopulation 1 germplasm had the highest yield per plant, which was 36.375 g. The yield per plant of the subpopulation 2 germplasm, domesticated in Japan, was the second highest, at 31.352 g, and the average yield per plant of germplasm domesticated in subpopulation 3 in China was the lowest, at 29.031 g.

Tajima’s D results showed that the NOSPP (Chr.3: 2,695,997–2,853,654) and 100-seed weight-related intervals (Chr.3: 6,058,466–6,182,360) located on chromosome 3 were both strongly selected (Figure S4). On chromosome 3, in the approximately 124 kb interval related to grain weight (Chr.3: 6,058,466–6,182,360), the average Tajima’s D of the subpopulation 2 germplasm of secondary domestication in Japan was 4.77181. However, the average Tajima’s D between the subpopulation 1 germplasm of Korea’s secondary domestication and the subpopulation 3 domestication of China were only −0.07481 and 1.36557, respectively. This suggests that balancing selection has been experienced in Japan due to the diversity of 100-seed weight-related alleles in this interval. However, in Korea and China, there is no evidence for screening for excellent alleles in this range. Balancing selection is a relatively rare form of positive selection. Usually, the occurrence of balanced selection in an interval indicates that there are different genotypes in this haploid that show advantages in different traits. The candidate genes in this interval are not only related to 100-seed weight, but also have other genotypes related to other important traits.

There are three possible candidate genes in this interval. The remorin-like protein, Grain setting defect1 (GSD1), affects the grain setting of rice grains by regulating plasmodesmata (PD) conductance [64,65]. MYB and Asp-Glu-Ala-Asp (DEAD)-box ATP-dependent RNA helicase 8-like among the candidate genes may not only affect grain size, but also may affect tolerance to abiotic stresses such as drought, salinity, and alkali. DEAD-box ATP-dependent RNA helicase 8-like may inhibit Group A protein phosphatases type 2C (PP2CA) activity to positively regulate ABA signaling, thereby regulating the response to drought stress. The rh8 knockout mutant exhibits ABA hyposensitivity and reduced drought tolerance [66]. In rice, it was found that R2R3MYB regulates grain weight by promoting the expression of brassinosteroids and the accumulation of sugars during seed development [67]. Moreover, the rice small grain mutant (SMG3) encodes a MYB-like protein that produces long grains by increasing the cell length and the number of cells along the lengths, thereby increasing the grain weight by promoting cell expansion and cell proliferation [68,69]. In soybean, R2R3MYB ensures grain weight by enhancing tolerance under a saline-alkali stress environment [70]. Therefore, the candidate genes in this interval may also be related to tolerance against abiotic stresses such as drought, salinity, and alkali.

The pairwise Fst between the three subpopulations reflects the differences in genotype frequencies (Figure S5). The total Fst of the three subpopulations only reaches 0.151 in this interval, which is only a moderate level of differentiation. However, the difference between the secondary domesticated subpopulation 1 in Korea and subpopulation 3, domesticated in China, remains at 0.5, which is a very large level of differentiation. Further, subpopulation 2, subpopulation 1, and subpopulation 3, domesticated in Japan, only have a medium level of differentiation (Fst = 0.209) and a small level of differentiation (Fst = 0.087). This shows that within the domestication selection in this interval, the Korean-domesticated subpopulation 1 and the Chinese-domesticated subpopulation 3 seem to have had different domestication goals. Therefore, we speculate that the genotype of Korean adzuki beans is biased in favor of grain weight. Although the genotypes of subpopulation 3, domesticated in China, retain genotypes that are beneficial to grain weight, the frequency of genotypes biased toward stress resistance is higher. Subpopulation 2, domesticated in Japan, has simultaneously conserved two genotypes with regard to stress resistance and grain weight via strong positive selection. The frequencies of the two genotypes are similar and they lack other rare alleles. Of course, alleles biased toward grain weight as well as abiotic stress resistance require experimental demonstration of gene function.

Our results are consistent with the growing environment of each origin. The climate in Korea is humid and adzuki beans have been conventionally grown on a small scale in relatively fertile fields since ancient times. In China, adzuki beans are often planted in mountains and on roadsides. These differences in planting environments may lead to differences in the direction of domestication. These conclusions are also consistent with the phenotypic trends of domesticated subpopulations in various origins. The variance analysis of the grain weights of the three subpopulations also showed that 100-seed weight of the Korean secondary domestication subpopulation had the heaviest grain weight, with an average of 14.187 g, whereas subpopulation 2, domesticated in Japan, was second, with an average weight of 100 grains of 11.903 g. The domesticated subpopulation 3 in China had the lowest 100-seed weight, with an average of only 10.284 g.

The 100-seed weight GWAS results showed that there were four SNPs in the range 9,757,052–10,654,699 in chromosome 3, exceeding the threshold (Table S3). In these four intervals, there are seven LURP-one-related proteins (LURP1). These include six protein LURP-one-related 15-like and one protein LURP-one-related 10-like. These genes are thought to be involved in resistance [71]. However, no candidate genes known to be related to grain weight were found in this interval. Those that may be related include TOR 1-like protein related to seed morphology and signal transducing adapter molecule 2-like related to signal transduction [72,73]. Tajima’s D showed that the Japanese-domesticated subpopulation 2 also strongly selected resistance genes and grain weight genes in this range. The peaks of balanced selection were all within the LURP1 gene interval. The highest peak occurred at 10,053,771–10,056,254 and Tajima’s D was 7.33763. In subpopulation 1, domesticated in Korea, selection was made only for 100-seed weight and the intensity of selection was not high. Tajima’s D level fluctuated between −1.33084 and −0.92423. The intensity of domestication selection in China is low, and LURP1 on 10,053,771 to 10,056,254 preserves the diversity of excellent genotypes related to 100-seed weight and resistance trait.

These results show that Korean breeders were more focused on domesticating selection for grain weight. Early breeders in China had less selective pressure on these two traits, while Japanese breeders tried their best to preserve the excellent genotypes for these two traits. In addition, both subpopulation 2 and subpopulation 3 conserved diversity between 10,053,771 and 10,056,254. The grain weight trait was always selected, but due to the recent strong selection event that has led to the elimination of rare alleles, the resistance genes may be of great significance to this screening event.

In the 100GW relevant interval (Chr.10: 32,679,443–33,489,503), the average Tajima’s D of subpopulation 1 of secondary domestication in Korea was −1.92815 (Table S4). The average Tajima’s D of subpopulation 2, domesticated in Japan, and subpopulation 3, domesticated in China, were 0.17452 and 3.0271, respectively. The 100-seed weight candidate gene section had introduced several functional genes in this range that may be related to the 100-seed weight: WRKY transcription factor 72, transcription factor MYB3-like, and basic leucine zipper 43-like. Stress- and defense-related genes include protein NSP-INTERACTING KINASE 1-like, hydroxyproline O-galactosyltransferase Galactose-1-Phosphate Uridylyltransferase (GALT) GALT6-like, an uncharacterized mitochondrial protein, tetratricopeptide repeat protein 38, three thioredoxin H2-like, Tobacco mosaic virus (TMV) resistance protein N-like, low quality protein: ultraviolet-B receptor UVR8, two tandem proteins NRT1/PTR FAMILY 2.13-like, alpha-amylase 3, chloroplastic-like, probable galacturonosyltransferase 9, putative calcium-transporting ATPase 13, plasma membrane-type, 6-phosphogluconate dehydrogenase, decarboxylating 3-like, single-stranded DNA-binding protein WHY1, two glutathione S-transferase U18-like, protein glutamine dumper 5-like, leaf rust 10 disease-resistance locus receptor-like protein kinase-like 1.5, and two stress- induced protein KIN2-like. NSP-interacting kinases (NIKs) included members involved in plant development and defense [74]. NIKs have been closely related to plant defense against geminiviruses. Numerous studies have confirmed its function in transducing defense signals against viral infection and its involvement in regulating brassinosteroid signaling, acting also as a regulator in developmental signaling pathways [74,75,76,77]. Arabinogalactan proteins (AGPs) play an important role in various growth and development and stress processes of plants [78]. Hydroxyproline O-galactosyltransferase mediates the biosynthesis of the polysaccharide Arabinogalactan. GALT6 mutants cause reduced root hair growth, reduced seed coat mucus, reduced seed setting, and accelerated leaf senescence. In addition, under salt stress, the root tips of the GALT6 mutant showed defective anisotropic growth, indicating that GALT6 has the function of preventing root tip swelling due to salt stress. The tetratricopeptide repeat (TPR) protein mediates a variety of growth and development, immune responses, and hormone-regulated signaling [79,80,81]. However, the specific function of tetratricopeptide repeat protein 38 is still unclear. Three thioredoxin H2-like proteins were found in this interval. The Trx-hs plays multiple roles in processes such as plant growth, development, stress response, and phytohormone signaling. The Trx-h2 protein transmits external cold stress signals to the downstream cold defense signaling cascade via its protein disulfide reductase function [82], allowing plants to exhibit cold tolerance characteristics. The TMV resistance N-like protein has been widely reported to be responsible for disease resistance in various plants [83,84,85,86]. Under ultraviolet B radiation stress, the Ultraviolet-B (UV-B) receptor UV resistance locus 8 (UVR8) promotes flavonoid biosynthesis, which acts as a sunscreen to enhance UV-B stress tolerance [87]. Members of the NRT1/PTR family (NPF) not only transport NO3, peptides, and amino acids, but also transport dicarboxylates, glucosinolates, indole-3-acetic acid and abscisic acid [88]. However, the specific function and mutant phenotype of protein NRT1/PTR family 2.13-like are still unclear. The α-amylase is related to hydrolyzing starch to produce glucose and maltose to mobilize stored energy [89]. Probable galacturonosyltransferase 9 is related to pectin biosynthesis, which is an important component of the cell wall. The Ca^2+^ pump ATPase plays a crucial role in maintaining Ca^2+^ homeostasis after different stimuli [90]. The 6-phosphogluconate dehydrogenase (6PGD) produces nicotinamide adenine dinucleotide phosphate (NADPH) by converting D-gluconolactone 6-phosphate to D-ribulose 5-phosphate in the pentose phosphate pathway (PPP) [91]. NADPH provides reducing equivalents to maintain homeostasis during oxidative stress [92,93,94]. The expression of Whirly (WHY) is strongly induced by Plasmopara viticola in grape plants. WHY1 mediates Pathogenesis-related (PR) protein expression and participates in the disease resistance responses. Transcriptional differences in the cell wall modification regulatory protein glutathione S-transferase U18-like proteins were found during the heat stress response as well as in the Fusarium wilt caused by Fusarium oxysporum in banana and lily [95,96]. The Glutamine dumper (GDU) is related to amino acid transport and controls amino acid homeostasis, as well as it may be involved in mediating drought and salt stress tolerance mechanisms [97]. Transcriptional defects in LRK10L1.2 cause plants to exhibit abscisic acid (ABA) insensitivity during seed germination and seedling growth. Moreover, a LRK10L1.2 mutant was found to have reduced tolerance to drought stress, indicating that LRK10L is a positive regulator of plant drought tolerance [98]. Stress-induced protein KIN2-like (KIN2) enhances plant resistance to salt, cold stress, and drought stress by enhancing plant sensitivity to ABA [99,100,101]. In addition, single-stranded WHY1 located in this interval is a disease resistance gene, and galacturonosyltransferase 9 is related to Growth, development and stress adaptability [102,103]. A high-frequency genotype was selected in the Korean-domesticated subpopulation 1 within this interval. In the Japanese-domesticated subpopulation 2, genotypes in this interval were not observed to be selected. This range of Chinese germplasm may have experienced domestication selection under strong biotic/abiotic pressures, with few rare alleles and a majority of neutral alleles. This means that in addition to grain weight, at least one stress resistance trait should have been selected. High-yielding genotypes and highly resistant genotypes are not consistent in this interval. Selection of resistant genotypes is often accompanied by the loss of high-yielding genotypes.

Within the relevant interval of NOSPP (Chr.3: 2,695,997–2,853,654), the Chinese-domesticated subpopulation 3 has experienced balancing selection, and Tajima’s D was 2.83125. Subpopulation 1, which was domesticated twice in Korea, and subpopulation 2, which was domesticated in Japan, have not been domesticated in this interval recently, and the average Tajima’s D was 0.36527 and −0.36911, respectively. However, this interval was not only mapped to NOSPP via GWAS, but traces of domestication selection were also found in subpopulation 3. We scanned each annotated candidate gene and found a large number of genes related to emergency response under stress. These genes include V-type proton ATPase subunit d2, thioredoxin H2, putative nuclease HARBI1, and heat shock 70 kDa protein 17. V-ATPase has been known to be involved in stress responses to various stresses including salt, cold, and heavy metals. Stress adaptability can be enhanced by overexpressing a certain V-ATPase subunit [104]. VHA-d2 plays a role in Arabidopsis thaliana’s response to oxidative stress [105]. Thioredoxin H2, has been reported to be responsible for cold tolerance, antioxidant defense, and improved smoke tolerance in different plants [82,106,107]. MdHARBI1 is significantly induced by heat stress, and its overexpression enhances tomato thermotolerance as well as autophagy under heat stress. In Arabidopsis thaliana, the heat shock protein (70 kDa) has been reported to be important for heat tolerance, and its mutation was lethal to female gametes as well as reduced the transmission efficiency of male gametes [108]. The average genetic differentiation index within this interval showed no differentiation between subpopulation 1 of secondary domestication in Korea and subpopulation 2 of secondary domestication in Japan, and Fst was only 0.013. The Chinese-domesticated subpopulation 3 and subpopulations 1 and 2 both had moderate genetic differentiation, with Fst being 0.160 and 0.230, respectively. Therefore, in the NOSPP-related interval on chromosome 3, only the Chinese-domesticated subpopulation 3 has undergone positive selection on NOSPP and emergency response-related genes.

In another NOSPP-related interval in chromosome 9 (Chr.9: 7,830,950–8,215,139), the Korean secondary domesticated subpopulation 1 and the Chinese-domesticated subpopulation 3 seem to have experienced strong selection, with average Tajima’s D of 2.79209 and 2.70773, respectively (Table S5). In subpopulation 2 of secondary domestication in Japan, the average Tajima’s D in this interval was −0.25717 and had not experienced selection. In this interval, in addition to cell division-related genes, there were also a large number of defense and systemic immunity genes. These genes included protein enhanced downy mildew 2-like, putative lipid-transfer protein Defective in induced resistance 1 (DIR1), sodium/hydrogen exchanger 4, and disease resistance protein RPM1-like. The EDM2 regulates the resistance of Ribonuclease P protein (RPP) RPP7 to the parasitic hyaline downy mildew isolate Hiks1 (HpHiks1) [109]. Systemic acquired resistance (SAR) is an induced defense response in plants in response to a local pathogen attack. In this defense response, DIR1 interacts with lipid-derived molecules to promote long-distance signaling to elicit systemic immunity [110,111]. In plants, sodium/hydrogen exchanger 4 confers alkaline salt tolerance to plants by regulating Na^+^/K^+^ homeostasis [112,113]. Maculicola 1 (RPM1) is an important immune receptor that enhances plant resistance to pathogenic bacteria [114,115,116]. The Japanese-domesticated subpopulation 2 had no trace of selection of these disease resistance genes and may have retained more abundantly high NOSPP alleles. This is also consistent with the Japanese secondary domestication subpopulation 2, the phenotype with the highest NOSPP. In addition, the genetic differentiation index showed that there was no obvious genetic differentiation between subpopulation 1, domesticated in Korea, and subpopulation 3, domesticated in China. The Japanese-domesticated subpopulation 3 and the other two subpopulations were shown to exhibit extreme (Fst = 0.295) and moderate (Fst = 0.220) genetic differentiation, respectively. And, within this interval, subpopulation 2 maintains higher genetic diversity.

Among the QTL associated with NOPPP (Chr.9: 25,969,508–26,273,940), subpopulation 2 of Japan’s secondary domestication showed in the interval 25,969,508–26,087,671 that it had experienced balancing selection in the recent past (Table S6). At least two different traits have experienced strong selection, whereas in the other two subpopulations, no traces of strong selection were found. There are 10 genes in this approximately 118 kb interval, seven of which are related to stress adaptation and defense, two genes are related to development and growth regulation, and one mediates both growth and stress defense. Development-related genes include serine/threonine-protein kinase lattice corneal dystrophy type I (CDL1)-like, the pentatricopeptide repeat-containing protein, and protein trichome birefringence-like 11. Resistance-related genes include DETOXIFICATION 40, Derlin-2.2, 15.4 kDa class V heat shock protein, beta-glucosidase 47-like, glucose-6-phosphate/phosphate translocator 2, peroxiredoxin-2F, mitochondrial-like. The serine/threonine-protein kinase CDL1-like protein positively regulates brassinosteroids (BR). The pentatricopeptide repeat-containing protein improves/prevents embryogenesis and plant development. Acetylation of cell wall polymers mediated by the trichome birefringence-like protein plays an important role in plant growth and stress defense [117,118]. Mutations in the trichome birefringence-like protein can cause dwarfism-like developmental dysplasia and show susceptibility to bacterial diseases [117,119]. Differential expression of DETOXIFICATION 40 (DTX40) in multidrug and toxic compound extrusion (MATE) efflux family proteins enhances adaptability to metal stresses such as aluminum and cadmium [46,120]. Derlin-2.2 is an oxidative stress program-related protein that helps the misfolded proteins to migrate to the cytosol for ubiquitination and eventual systemic degradation [121,122]. The 15.4 kDa class V heat shock protein accumulates under heat stress, controls signaling, protein translocation, and degradation, as well as alleviates the negative effects of heat stress by preventing protein misfolding and aggregation and protects cell membranes [123,124]. Sugars such as glucose have been reported to aid plants by improving their cold tolerance [125,126]. Lysosomal beta-glucosidase 47-like is up-regulated during low temperature stress and is used to synthesize glucose and resist low temperature stress [127]. Glucose-6-phosphate/phosphate translocator 2 (GPT2) is involved in environmental sensing including sugar signals, light signals, etc., and may be used to maintain the cytoplasmic redox balance [128,129]. Mitochondrial peroxiredoxin-IIF (PRXIIF) is a thiol peroxidase with an important role in protecting cells by sensing oxidative stress that occurs during disturbances in redox homeostasis [130,131]. Fst shows that in this interval, subpopulation 2, which focuses on stress adaptability and defense, has great genetic differentiation from subpopulations 1 and 3, domesticated in Korea and China, whose Fst were 0.369 and 0.313, respectively.

Subpopulation 1 of secondary domestication in Korea showed that it experienced different balancing selection than subpopulation 2 in the interval 26,130,125–26,227,726. Just like the balancing selection in the NOSPP correlation interval, domestication selection for disease resistance and stress adaptability may cause the death of a large number of individuals in the population and affect allele frequencies, while domestication selection for yield traits is a long-term behavior. Stresses such as disease and drought may cause the death of a large number of individuals. The frequency of alleles with excellent stress adaptability increases, while high-yield alleles become rare alleles and are later re-selected. Genes related to growth and development include neurogenic protein mastermind-like (MAML), chloroplastic adenylate isopentenyltransferase 5, and mitochondrial pentatricopeptide repeat-containing protein At5g19020, of which resistance-related genes include nuclear transcription factor Y subunit C-3-like, dihydroxy-acid dehydratase, chloroplastic, basic 7S globulin (Bg7S)-like proteins, transcription factor MUTE-like, and serine incorporator 3. Neurogenic protein mastermind-like is considered to be a component of developmental signaling pathways and plays an important role in embryonic development, determining cell fate, and late cell differentiation [132]. The adenylate isopentenyltransferase 5, chloroplastic-like is a cytokinin biosynthetic enzyme. Pentatricopeptide repeat-containing protein (PPR) proteins play roles in transcription, RNA processing, splicing, stability, editing, and translation. PPR proteins are important for organelle genome expression and organelle biogenesis. Mutants of these proteins often exhibit embryonic developmental defects and cytoplasmic male sterility [133]. Nuclear transcription factor Y subunit C-3-like (NF-YC) is one of the subunits that make up the Nuclear Factor-Y. The NF-Y complex regulates the expression cassette of target genes by directly binding to the promoter CCAAT or by physically interacting and mediating the binding of transcriptional activators or inhibitors. NF-Y not only regulates plant growth and development, but also plays an important role in abiotic stress responses such as drought, salt, nutrients, and temperature [86]. The dihydroxy-acid dehydratase, chloroplastic (DHAD), is an autoresistance gene [134]. DHAD is involved in the biosynthesis of the plant toxin branched-chain amino acid. Therefore, it is also used as a target for genetic modification of natural herbicides. The basic 7S globulin (Bg7S)-like proteins are a major seed storage protein but are involved in stress response, antimicrobial activity, and hormone receptor-like activity [135]. Plant pathogenic microorganisms synthesize glycosyl hydrolase, which digests the polysaccharides that constitute the plant cell wall and enters the cell. Xyloglucan-specific endo-β-1,4-glucanase (XEG) is one of the glycosyl hydrolases used by pathogenic microorganisms to attack plant cells. Bg7S specifically inhibits XEG and resists the digestion of cell walls by pathogenic microorganisms. The transcription factor MUTE-like is a gene for stomatal development in leaves under drought stress. The regulation of drought stress response in plants reduces the expression of MUTE-like bHLH and downregulates stomatal density to improve drought tolerance [136]. The serine incorporator 3 is involved in the virus’s defense response. Fst shows that subpopulation 1′s stress adaptability and defense genotypes were selected to have great genetic differentiation with subpopulations 2 and 3, domesticated in Japan and China, with Fst being 0.392 and 0.257, respectively. The domesticated subpopulations 2 and 3 in Japan and China did not show strong selection and there was no genetic differentiation between the two subpopulations, with an Fst of 0.036. The NOPPP phenotype of domesticated subpopulation 3 in China is the highest, which is consistent with the conclusion drawn in the genetic diversity analysis. No strong domestication selection was observed in subpopulation 3, domesticated in China, so we believe that subpopulation 3 retained more high-yield alleles within these two intervals. In the domesticated subpopulations 1 and 2 of Korea and Japan, resistance and stress adaptability genes were strongly selected in two intervals, respectively, and part of the genotypes related to high yield may have been lost in the process.

According to the traces of domestication selection reflected in the diversity data of yield-related QTL, it was found that the differences in three yield-related traits (100GW, NOSPP, NOPPP) among domesticated subpopulations in different regions are not completely related to the domestication selection preferences of early breeders and farmers. Planting habits, planting environment, and historical events (biotic and abiotic stresses) that were experienced seems to have a greater impact on the yield-related traits of each subpopulation. In our results, a large number of biotic/abiotic stress adaptation genes were found in LD blocks for yield-related traits. We discussed the impact of various stress-resistant selections on the production of yield traits in historical events. In the modern adzuki bean breeding process in recent years, a strong selection of yield-related genes has also caused background selection, which will reduce the diversity of these resistance genes. This will limit genetic diversity and stress resistance within elite varieties, threatening the safety of adzuki bean production. We believe that at this stage, we need to actively carry out diversification of cultivated varieties and protection of germplasm resources to cope with various natural risks and ensure food security.

## 4. Materials and Methods

### 4.1. Plant Material

A total of 366 adzuki bean Landraces with their origins in China (96), Japan (119), and Korea (151) currently conserved at The National Agro-biodiversity Center of the Rural Development Administration (RDA), Republic of Korea were taken for studies. All adzuki bean germplasm were planted in experimental fields in Jeonju City, South Korea (35°49′50.952″ N, 127°3′44.604″ E) from June to October 2021. Each landrace was planted with 10 biological replicates, with rows spaced 90 cm apart and plants 15 cm apart. Investigations were carried out for recording 11 agronomic traits (including bottom pod height, flowering date, maturity date, growth period, plant height, lodging score, synchronous maturing, 100-seed weight, number of pods per plant, number of seeds per pod, and yield) related to domestication. The actual measurement numbers were recorded for 11 quantitative traits, and the data for each landrace was the average of 10 biological replicates. The date when the five plants bloomed was recorded as the flowering date, and the percentage of mature pods in the number of pods was observed three days after the first pod matured.

### 4.2. DNA Extraction

Six weeks after sowing, young leaves of 366 adzuki bean landraces were sampled, freeze-dried, and stored at 4 °C. Genomic DNA from leaf tissues was extracted using the QIAGEN DNeasy^®^ Plant Mini Kit (Hilden, Germany). The concentration and quality of DNA was measured using a UVS-99 UVSDrop spectrophotometer (ACTGene, Piscataway, NJ, USA). After diluting the DNA to 100 ng/μL, the DNA quality was verified once more on a 1% agarose gel and stored at −20 °C.

### 4.3. Genotyping by Sequencing

DNA was digested with the Apek I enzyme and the adapters for labeling were ligated to create a GBS library. A total of 366 adzuki bean landraces were sequenced in one lane using Illumina HiSeq X (Illumina, San Diego, CA, USA). SolexaQA v.1.13 [137] was used to perform read alignment and error correction. SNP calling was based on the Vigan1.1 [138] reference genome. The SNPs were filtered using Tassel 5 [139]. The filtering settings were maximum major allele frequency = 0.95, maximum missing data = 0.05, maximum heterogeneous proportion = 0.1; filter individual: maximum heterogeneous proportion = 0.1. The SNPs that were not assembled onto the chromosome were manually filtered.

### 4.4. Genome-Wide Association Study

Genome-wide association studies were performed using the mixed linear model (MLM) of the GAPIT3 R package (version 3.4.0) [140] and the results were visualized using the CMPlot R package (version 4.5.0). The haplotype blocks associated with agronomic traits were calculated using the confidence interval model of Haploview v4.2 [141] and used to determine the range of candidate genes.

### 4.5. Genetic Diversity and Differentiation Statistics

First, we conducted a population structure analysis on 366 adzuki bean landraces. Based on the genotype data of 6586 SNPs, Structure software (version 2.3.4) [142] was used to calculate the population structure with K values ranging from 2 to 10. Structure Harvester software [143] was used to calculate the optimal K value, which indicates the number of subpopulations. PLINK [144] was used to conduct the principal component analysis of genetic characteristics (6586 SNPs) of the 366 adzuki bean germplasms. In order to understand the diversity and domestication selection depth of each subpopulation and compare the diversity abundance of adzuki bean and other crops, the linkage disequilibrium decay distance of the adzuki bean population as a whole and each subpopulation was calculated. First, Tassel 5 [139] was used to perform linkage disequilibrium analysis and the LD index table of pairwise SNPs was derived. The average LD decay distance was calculated and visualized using R v4.1.0. Genomic sliding window Tajima’s D was calculated using Tassel 5 [139], with both the step size and window size set to 3. The genome-wide genetic differentiation index (Fst) between each subpopulation was analyzed in GenAlEx 6.5 [145]. A rooted phylogenetic tree was constructed using neighbor-joining (NJ) models in Tassel 5 [139] and visualized in iTOL [146]. Three accessions each of rice bean, cowpea, and mung bean, closely related species of adzuki bean, were introduced as outgroups to determine the oldest adzuki bean germplasm with the closest genetic distance to the outgroup. Migrate-n v5.0.4 software [147] was used to estimate gene flow between the three subpopulations distributed in the three origins based on the Brownian motion model.

### 4.6. Statistical Analysis

Descriptive statistics and ANOVA on agronomic traits of each subpopulation were performed using XLSTAT trial version (Microsoft Corporation, Charlotte, NC, USA).

## 5. Conclusions

In this study, three adzuki bean subpopulations obtained via population structure analysis were specifically distributed in three regions of Korea, China, and Japan. There were significant differences in three yield traits (number of pods per plant, number of seeds per pod, and 100-seed weight) among the three subpopulations. QTLs for these three traits were mapped via GWAS. Genomic sliding window Tajima’s D and the genetic differentiation coefficient showed the differences in domestication selection intensity and allelic diversity in yield-related QTL in different subpopulations. Tajima’s D showed that more than one genotype was selected. Gene function analysis in QTL showed that there are also a large number of stress adaptability genes within the interval. Tajima’s D and haplotype analysis confirmed that these genes were selected and caused background selection. The haplotype analysis results showed that the selection of stress resistance genes affected the yield-related genes linked to them, resulting in a reduction in the diversity of yield-related genes. Therefore, the differences in adzuki bean yield traits in the three regions of Korea, China, and Japan are not only caused by the preferences of early breeders, but adversity may have a greater impact on these differences.

## Figures and Tables

**Table 1:** Analysis of variance (ANOVA) showed differences in agronomic traits among subpopulations.

Traits	Value	Subpopulation			Skewness	Kurtosis
		1 (KOR)	2 (JPN)	3 (CHN)		
Number of pods per plant	Region	8.667–117.333	9.667–116	11.667–85.667	1.286	2.547
	Mean ± SD	39.351 ± 17.184 ^ab^	36.194 ± 19.272 ^b^	42.345 ± 14.507 ^a^		
	CV	0.437	0.532	0.343		
Number of seeds per pod	Region	4.4–9.5	4.4–9.9	4.6–9	0.140	−0.365
	Mean ± SD	6.678 ± 0.99 ^b^	7.178 ± 0.991 ^a^	6.889 ± 1.054 ^b^		
	CV	0.148	0.138	0.153		
100-seed weight (g)	Region	4.5–21.467	3.8–19.667	3.567–15.9	−0.010	−0.503
	Mean ± SD	14.187 ± 3.192 ^a^	11.903 ± 3.685 ^b^	10.284 ± 2.576 ^c^		
	CV	0.225	0.310	0.250		
Yield per plant (g)	Region	5.730–110.386	4.839–130.021	8.991–62.230	1.533	4.196
	Mean ± SD	36.375 ± 16.134 ^a^	31.352 ± 21.522 ^b^	29.031 ± 11.192 ^b^		
	CV	0.444	0.686	0.386		
Flowering date (DAS)	Region	47–77	44–77	47–76	−0.214	−0.778
	Mean ± SD	63.466 ± 6.53 ^a^	61.054 ± 8.468 ^b^	60 ± 7.488 ^b^		
	CV	0.103	0.139	0.125		
Maturity date (DAS)	Region	92–126	83–127	87–120	−1.093	2.568
	Mean ± SD	109.211 ± 4.998 ^a^	106.838 ± 6.546 ^b^	106.929 ± 6.446 ^b^		
	CV	0.046	0.061	0.060		

^a, b, c^ represent significantly different average values across a row (*p* < 0.05).

## Data Availability

Data are contained within the article and Supplementary Materials.

## Supplementary Materials

The following supporting information can be downloaded at: https://www.mdpi.com/article/10.3390/plants12244154/s1, Figure S1: Linkage disequilibrium (LD) decay plot of 366 adzuki bean germplasms as a total and each subpopulation. The green font shows the LD decay distance of each subpopulation; Figure S2: Box plots and scatter plots show the mean and distribution differences in yield-related traits among different adzuki bean subpopulations, respectively; Figure S3: Rooted phylogenetic tree of 366 adzuki bean landraces drawn using 6586 SNPs. Three accessions each of mung bean, cowpea, and rice bean were added to the phylogenetic tree as outgroups. The colors of label and branch represent each subpopulation. Each country of origin is marked in the Color strip using different colors. The three outer rings from the inside to the outside respectively display the number of pods per plant (NOPPP), the number of seeds per pod (NOSPP), and the 100-seed weight (100GW) of the corresponding germplasm; Figure S4: Among each QTL, Tajima’s D of each subpopulation showed different strengths of domestication selection; Figure S5: In each QTL, the genetic differentiation coefficient between each subpopulation represents the genotypic differences resulting from different domestication selections; Table S1: Diversity index of each subpopulation; Table S2: Migration rate between each subpopulation in all origins; Table S3. List of candidate genes in QTL for 100-seed weight on chromosome 3 (9,757,052-10,654,699), differentiation index between subpopulations in QTL and Tajima’ D of each subpopulation; Table S4. List of candidate genes in QTL for 100-seed weight on chromosome 10 (32,679,443-33,489,503), differentiation index between subpopulations in QTL and Tajima’ D of each subpopulation; Table S5. List of candidate genes in QTL for number of seeds per pod on chromosome 9 (7,830,950-8,442,610), differentiation index between subpopulations in QTL and Tajima’ D of each subpopulation; Table S6. List of candidate genes in the QTL for number of pods per plant on chromosome 9 (25,969,508-26,273,940), differentiation index between subpopulations in the QTL and Tajima’ D of each subpopulation. References [148,149,150,151,152,153,154,155,156,157,158,159,160,161,162] are cited in the Supplementary Materials.
