# Supplementary material for: Analysis of Genetic Diversity in Adzuki Beans (Vigna angularis): Insights into Environmental Adaptation and Early Breeding Strategies for Yield Improvement
Source: Plants (Basel). 2023 Dec 13;12(24):4154. doi: 10.3390/plants12244154 (PMC10747723; doi:10.3390/plants12244154)
Supplement: Supplementary file 1 [file plants-12-04154-s001.zip › Supplementary Figure & Table.pdf]

Supplementary Materials:

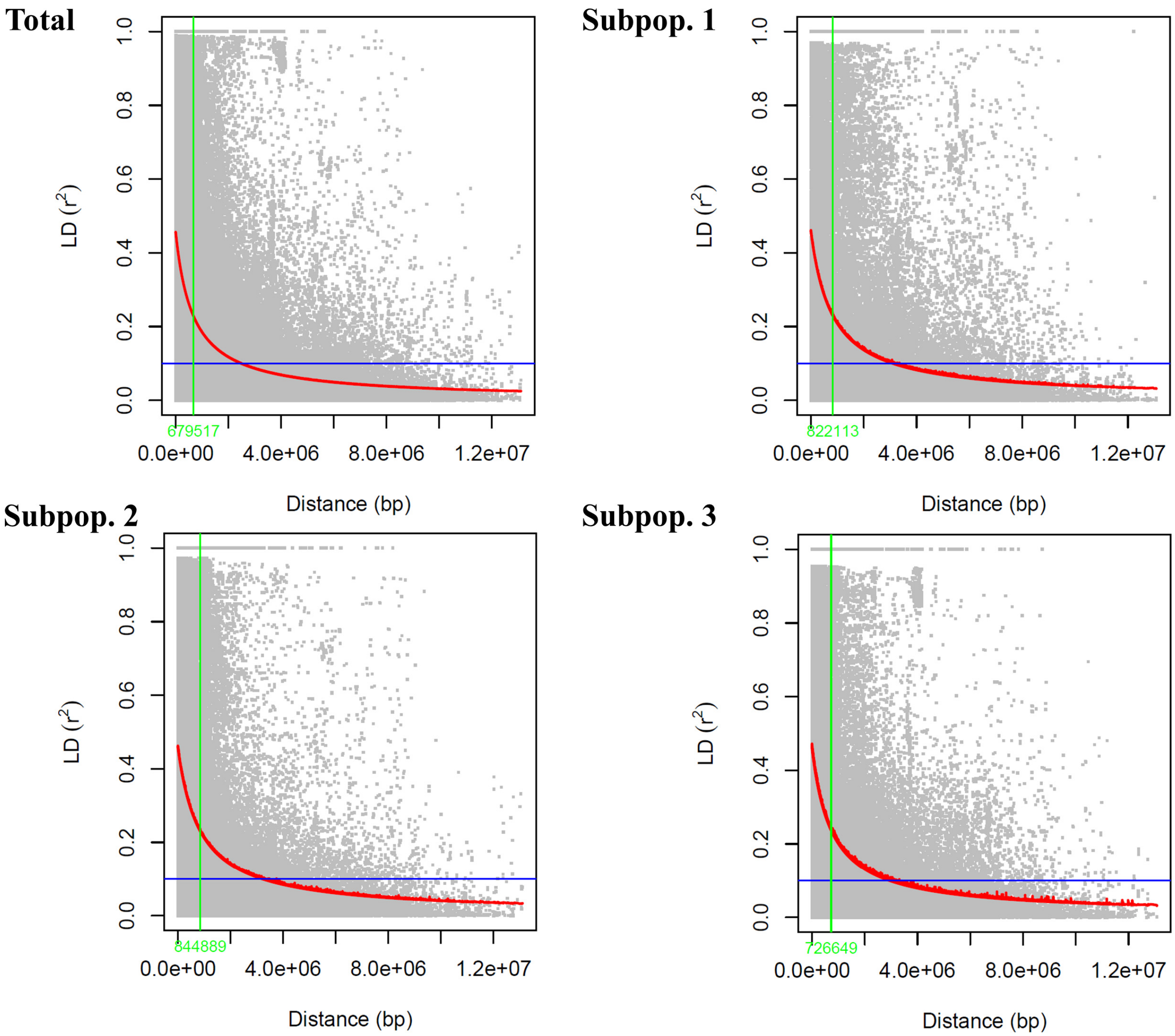

Figure S1. Linkage disequilibrium (LD) decay plot of 366 adzuki bean germplasm as a total and each subpopulation. The green font shows the LD decay distance of each subpopulation.

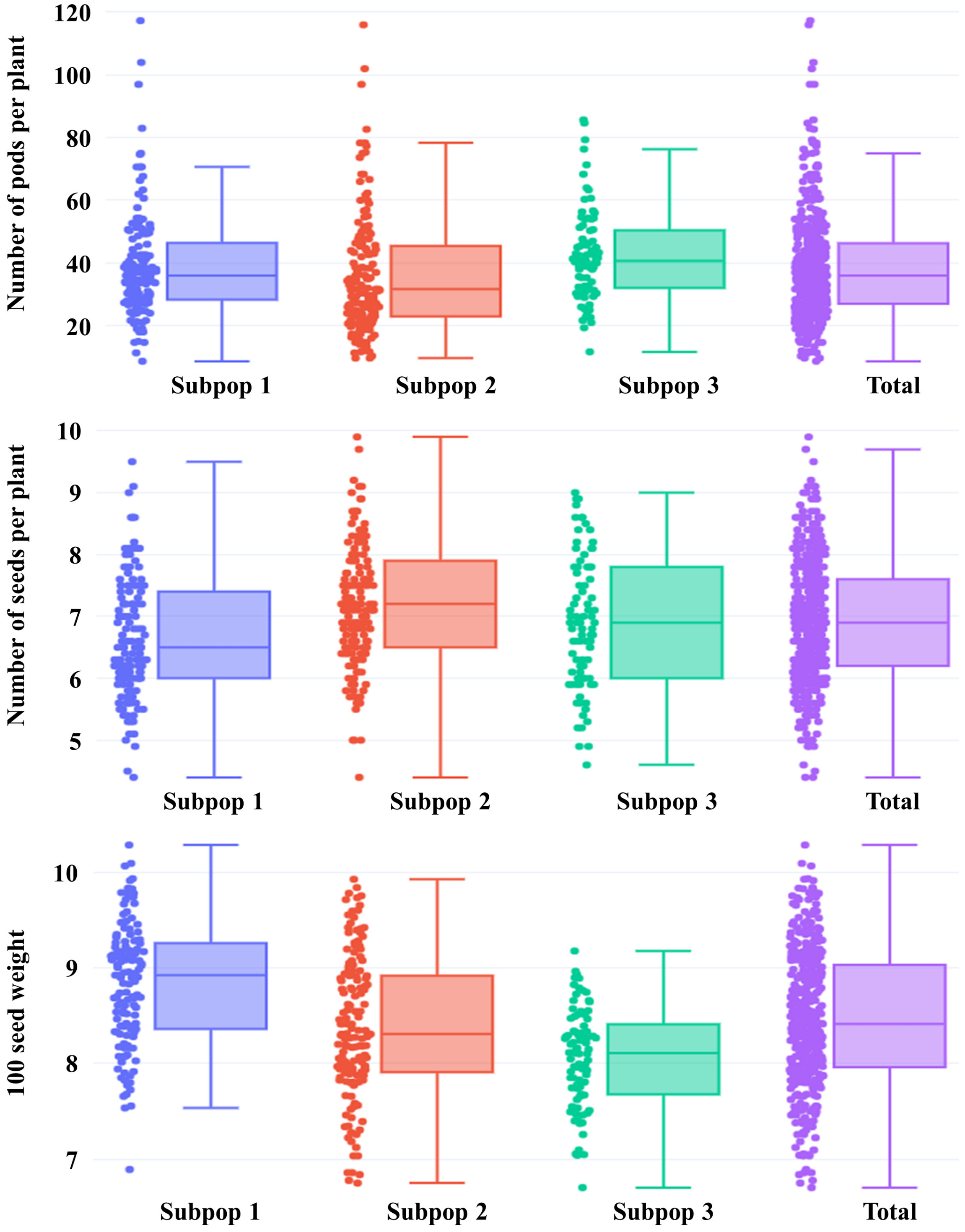

Figure S2. Box plots and scatter plots show the mean and distribution differences in yield-related traits among different adzuki bean subpopulations, respectively.

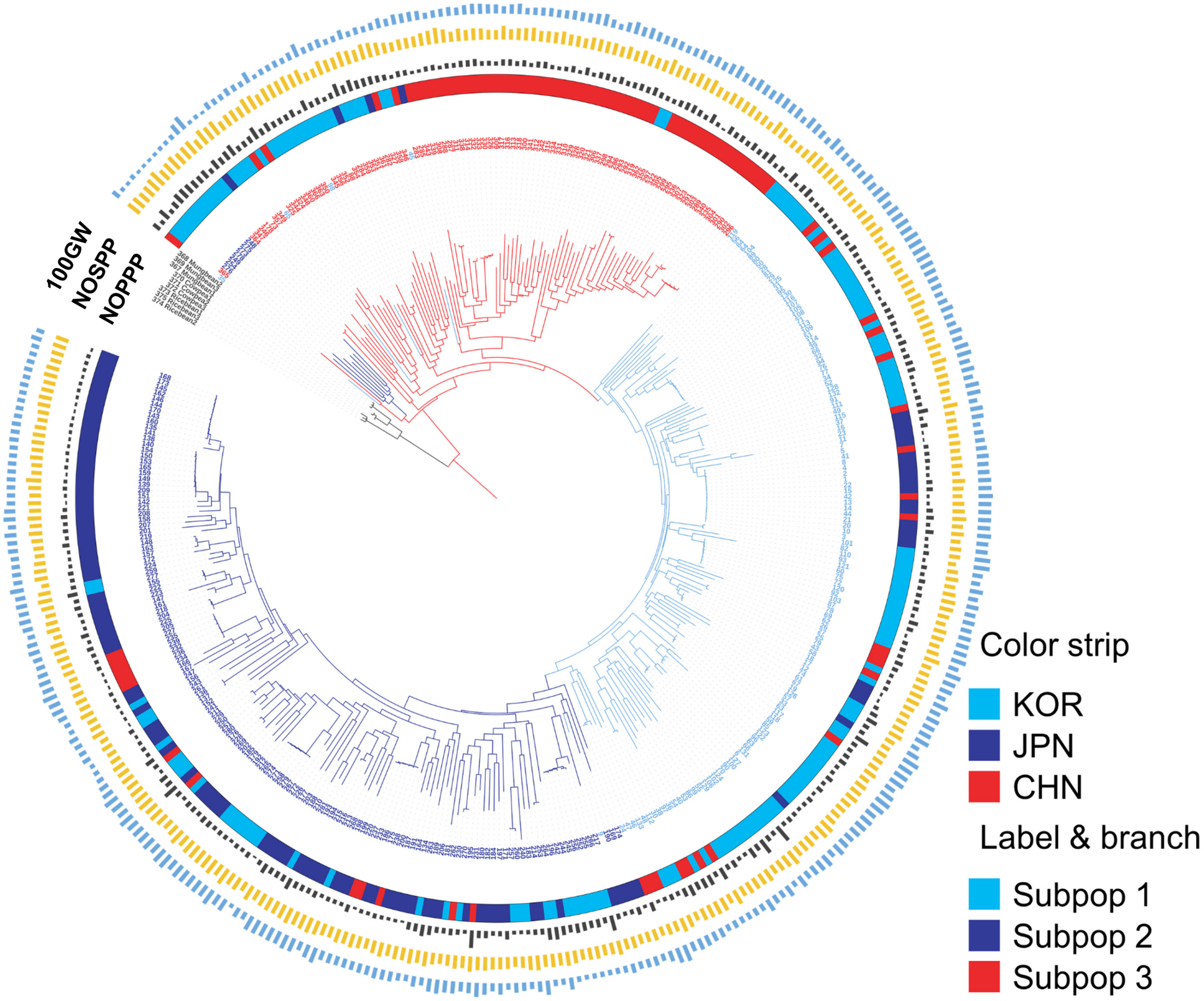

Figure S3. Rooted phylogenetic tree of 366 adzuki bean landraces drawn using 6586 SNPs. Three accessions each of mung bean, cowpea, and rice bean were added to the phylogenetic tree as outgroups. The colors of label and branch represent each subpopulation. Each country of origin is marked in the Color strip using different colors. The three outer rings from the inside to the outside respectively display the number of pods per plant (NOPPP), the number of seeds per pod (NOSPP) and the 100-seed weight (100GW) of the corresponding germplasm.

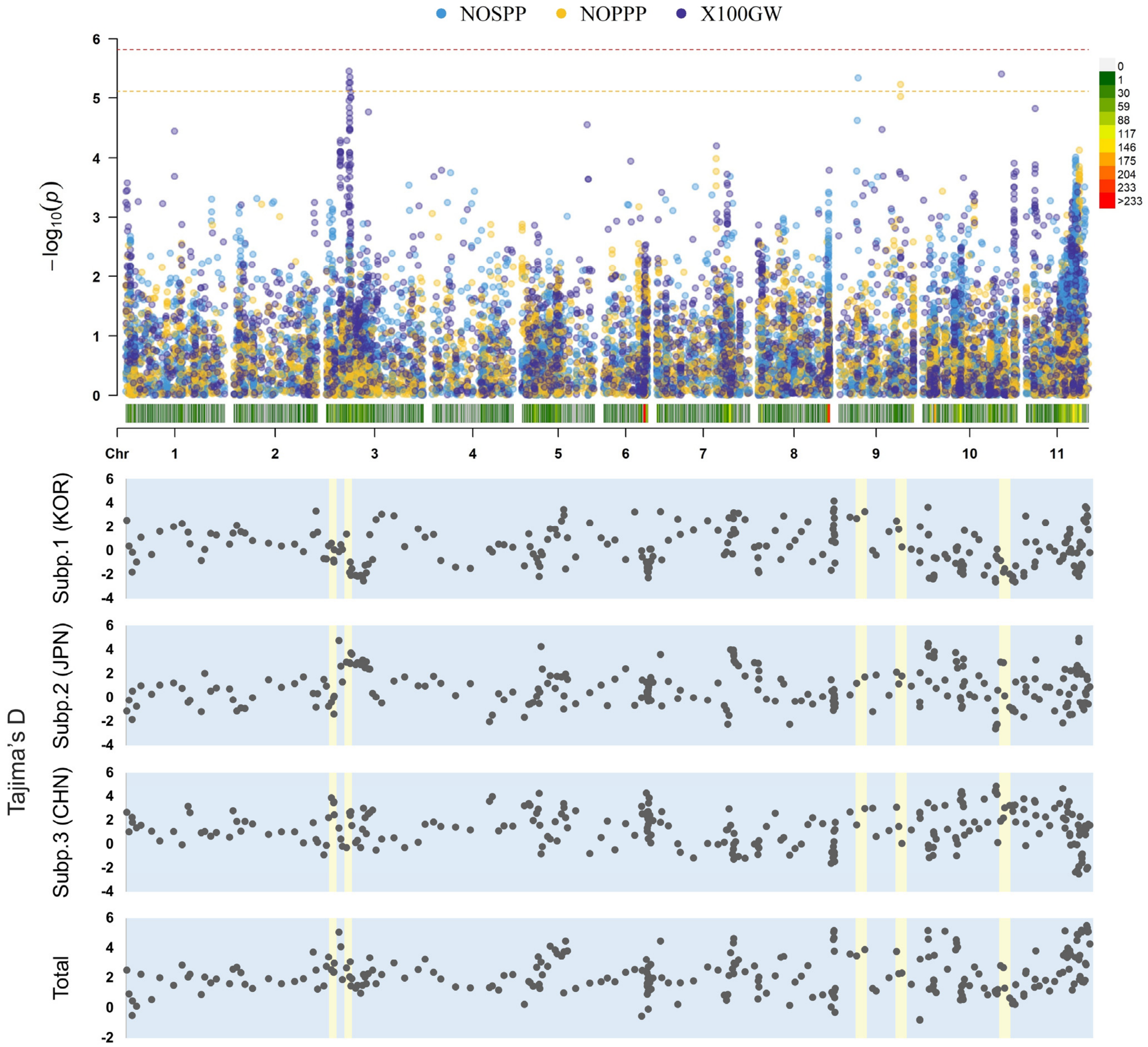

Figure S4. Among each QTL, Tajima's D of each subpopulation showed different strengths of domestication selection.

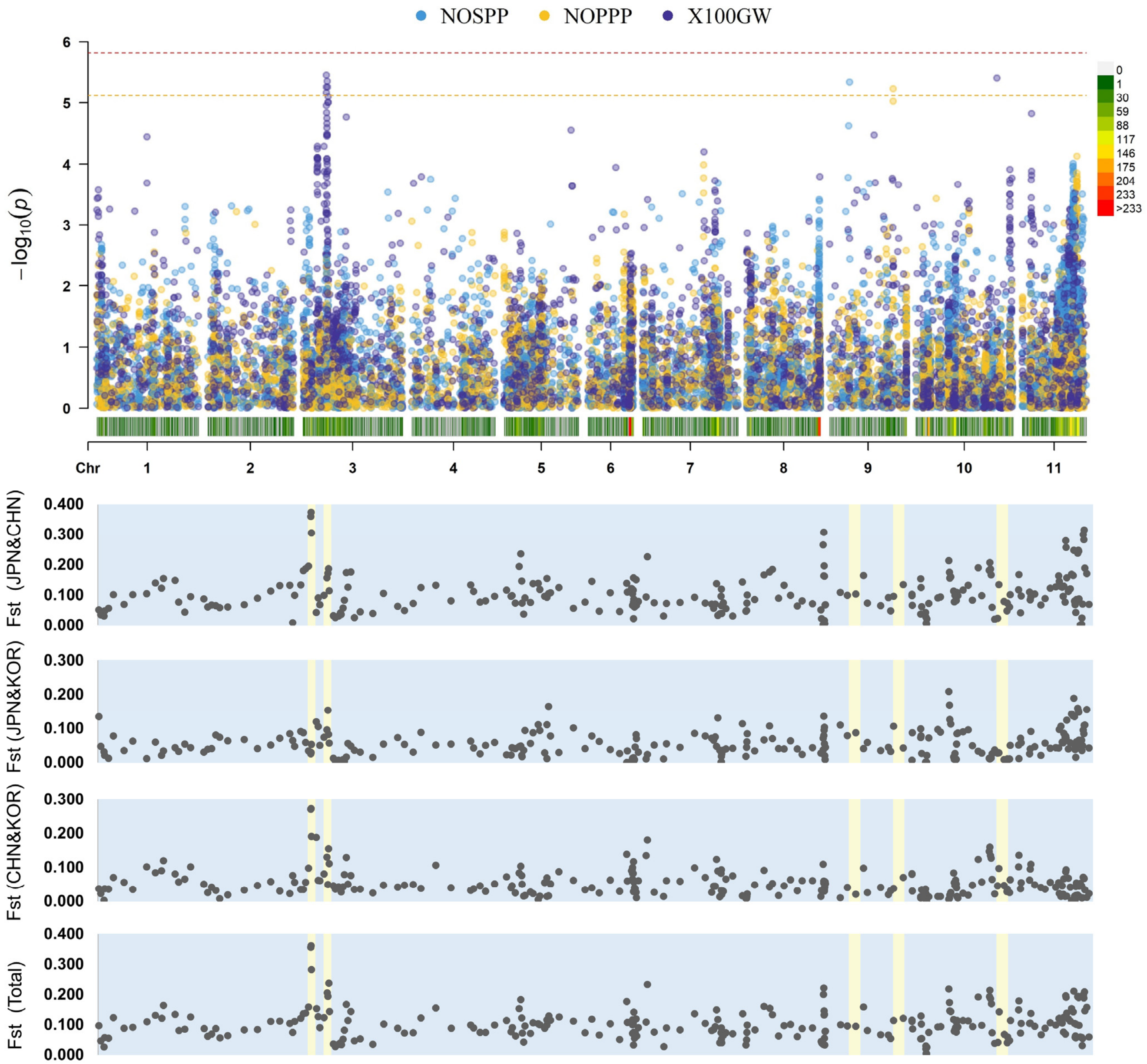

Figure S5. In each QTL, the genetic differentiation coefficient between each subpopulation represents the genotypic differences resulting from different domestication selections.

Table S1. Diversity index of each subpopulation.

**Subpop.**	**Na**	**Ne**	**I**	**Ho**	**He**
1	1.988±0.001	1.39±0.004	0.391±0.002	0.005±0	0.246±0.002
2	1.998±0.001	1.466±0.004	0.455±0.002	0.006±0	0.292±0.002
3	1.959±0.002	1.4±0.004	0.396±0.003	0.004±0	0.251±0.002
Total	1.982±0.001	1.419±0.002	0.414±0.001	0.005±0	0.263±0.001

Na, number of different alleles; Ne, number of effective alleles; I, Shannon's information index; Ho, observed heterozygosity; He, expected heterozygosity.

Table S2. Migration rate between each subpopulation in all origins.

Migration direction		Migration rate	Migration direction		Migration rate
From	To	(Mean)	From	To	(Mean)
KOR3	CHN1	111.4	**CHN1**	**KOR3**	**152.5**
**CHN3**	**CHN2**	**96.8**	CHN2	CHN3	49.3
**JPN2**	**CHN3**	**98.1**	CHN3	JPN2	69.3
**CHN2**	**JPN1**	**119.4**	JPN1	CHN2	67.3
**CHN2**	**JPN2**	**105.7**	JPN2	CHN2	77.4
**CHN2**	**KOR1**	**131.1**	KOR1	CHN2	77.1
**JPN1**	**KOR2**	**130.9**	KOR2	JPN1	45.9
**JPN2**	**KOR3**	**173.6**	KOR3	JPN2	104.8

Bold represents the gene flow direction with higher migration rate.

Table S3. List of candidate genes in QTL for 100-seed weight on chromosome 3 (9,757,052-10,654,699), differentiation index between subpopulations in QTL and Tajima' D of each subpopulation.

			Fst				Tajima' D			
**Name**	**Chr.**	**Position**	**pop1 &2**	**pop1 &3**	**pop2 &3**	**all**	**pop1 (KOR)**	**pop2 (JPN)**	**pop3 (CHN)**	**Function**
Protein LURP-one-related 10-like	3	9,768,231..9,769,903	0.242	0.098	0.044	0.188	-1.036	2.951	1.253	Resistance [71]
Hypothetical protein	3	9,788,712..9,791,039	0.261	0.005	0.223	0.220	-1.107	1.599	0.167	-
Uncharacterized protein	3	9,791,722..9,792,900	0.261	0.005	0.223	0.220	-1.107	1.599	0.167	-
Hypothetical protein and KOM37390.1	3	9,792,972..9,793,391	0.261	0.005	0.223	0.220	-1.107	1.599	0.167	-
Uncharacterized protein	3	9,794,661..9,796,304	0.261	0.005	0.223	0.220	-1.107	1.599	0.167	-
Hypothetical protein	3	9,800,472..9,801,652	0.143	0.005	0.115	0.121	-1.107	1.599	0.167	-
Protein LURP-one-related 15-like	3	9,800,481..9,801,669	0.024	0.006	0.007	0.023	-1.107	1.599	0.167	Resistance [71]
Uncharacterized protein	3	9,865,853..9,866,964	0.000	0.129	0.123	0.028	-1.049	-1.258	2.222	-
Protein LURP-one-related 15-like	3	9,919,645..9,920,773	0.259	0.010	0.201	0.223	-1.331	2.912	-1.016	Resistance [71]
Protein kinase 2A, chloroplastic-like	3	9,928,769..9,929,719	0.263	0.013	0.197	0.223	-1.331	2.875	-1.016	-
Protein LURP-one-related 15-like	3	9,957,918..9,958,859	0.267	0.017	0.193	0.224	-1.483	2.875	-1.016	Resistance [71]
Protein LURP-one-related 15-like	3	9,988,610..9,989,866	0.249	0.024	0.167	0.200	-1.314	2.976	-0.996	Resistance [71]
Uncharacterized protein	3	10,007,919..10,008,630	0.272	0.010	0.214	0.245	-1.345	-0.906	-0.996	-
Protein LURP-one-related 15-like	3	10,053,771..10,056,254	0.256	0.010	0.198	0.213	-0.924	7.338	2.785	Resistance [71]
Signal transducing adapter molecule 2-like	3	10,159,689..10,165,246	0.130	0.065	0.176	0.137	-1.160	1.912	1.942	Signaling [72]
TOR 1-like	3	10,186,060..10,203,485	0.189	0.061	0.163	0.166	-1.074	2.560	1.022	Seed morphology [73]
Alanine glyoxylate aminotransferase 2	3	10,238,885..10,244,500	0.011	0.123	0.156	0.064	-1.188	2.026	0.098	-
Protein LURP-one-related 15-like	3	10,567,789..10,569,748	0.253	0.002	0.225	0.214	-1.167	1.866	0.998	Resistance [71]
Uncharacterized protein	3	10,607,205..10,607,699	0.011	0.113	0.147	0.062	-1.167	1.887	0.387	-
Uncharacterized protein	3	10,653,890..10,655,477	0.144	0.030	0.060	0.022	-1.384	1.887	0.387	-

Table S4. List of candidate genes in QTL for 100-seed weight on chromosome 10 (32,679,443-33,489,503), differentiation index between subpopulations in QTL and Tajima' D of each subpopulation.

			Fst				Tajima' D			
**Name**	**Chr.**	**Position**	**pop1 &2**	**pop1 &3**	**pop2 &3**	**all**	**pop1 (KOR)**	**pop2 (JPN)**	**pop3 (CHN)**	**Function**
Stress-induced protein 2-like	10	32,685,837..32,686,293	0.010	0.157	0.188	0.069	-1.160	-0.389	2.514	Salt, cold and drought stress adaptability [99] [100] [101]
Stress-induced protein 2-like	10	32,690,138..32,690,594	0.010	0.157	0.188	0.069	0.670	-0.723	1.416	Salt, cold and drought stress adaptability [99] [100] [101]
NSP-intracting kinase 1- like	10	32,717,436..32,724,696	0.010	0.157	0.188	0.069	0.670	-0.723	1.416	Development and defense [74] [75] [76] [77]
Glutamine dumper 5- like	10	32,753,289..32,753,859	0.010	0.157	0.188	0.069	0.670	-0.723	1.416	Drought and salt stress adaptability [97]
GALT6-like	10	32,764,589..32,769,265	0.010	0.157	0.188	0.069	0.670	-0.723	1.416	Growth, development and stress adaptability [78]
U18-like	10	32,777,281..32,778,635	0.010	0.157	0.188	0.069	0.670	-0.723	1.416	Heat stress adaptability [95] [96]
U18-like	10	32,789,066..32,790,180	0.010	0.157	0.188	0.069	0.670	-0.723	1.416	Heat stress adaptability [95] [96]
Protein AtMg00810-like	10	32,803,066..32,805,128	0.010	0.157	0.188	0.069	0.670	-0.723	1.416	-
Tetratricopeptide repeat 38	10	32,817,910..32,823,560	0.010	0.157	0.188	0.069	0.670	-0.723	1.416	Growth, development and stress adaptability [79] [80] [81]
LRK10	10	32,823,619..32,826,077	0.010	0.157	0.188	0.069	0.670	-0.723	1.416	Drought tolerant [98]
TMV resistance protein N-like	10	32,887,149..32,888,190	0.010	0.157	0.188	0.069	0.670	-0.723	1.416	Disease resistance [83] [84] [85] [86]
TMV resistance protein N-like	10	32,890,778..32,891,903	0.010	0.157	0.188	0.069	0.670	-0.723	1.416	Disease resistance [83] [84] [85] [86]
Thioredoxin H2-like	10	32,904,675..32,905,626	0.010	0.157	0.188	0.069	0.670	-0.723	1.416	Growth and development, stress response [82]
Thioredoxin H2-like	10	32,909,522..32,910,626	0.010	0.157	0.188	0.069	0.670	-0.723	1.416	Growth and development, stress response [82]
Thioredoxin H2-like	10	32,922,711..32,923,528	0.010	0.157	0.188	0.069	0.670	-0.723	1.416	Growth and development, stress response [82]
Protein NRT1/ PTR 2.13-like	10	32,976,615..32,981,053	0.010	0.157	0.188	0.069	0.670	-0.723	1.416	Growth regulatory/transport hormone [88]
Ultraviolet-B receptor UVR8	10	33,000,179..33,007,024	0.010	0.157	0.188	0.069	0.670	-0.723	1.416	UV stress adaptability [87]
Protein NRT1/ PTR FAMILY 4.6	10	33,080,526..33,085,567	0.025	0.101	0.205	0.037	0.670	-0.723	1.416	Growth regulatory/transport hormone [88]
Aldehyde oxidase GLOX	10	33,096,639..33,098,328	0.025	0.101	0.205	0.037	0.670	-0.723	1.416	-
Probable receptor-like	10	33,162,509..33,166,605	0.025	0.101	0.205	0.037	0.670	-0.723	1.416	-
Alpha-amylase 3-like	10	33,202,852..33,209,856	0.025	0.101	0.205	0.037	0.670	-0.723	1.416	Store energy [89]
Probable WRKY TF 72	10	33,298,511..33,300,526	0.083	0.010	0.126	0.024	0.670	-0.723	1.416	100 seed weight [40] [41]
Single-stranded WHY1	10	33,343,138..33,345,272	0.083	0.010	0.126	0.024	0.670	-0.723	1.416	Disease resistance [102]
ATPase 13	10	33,394,845..33,398,586	0.083	0.010	0.126	0.024	0.670	-0.723	1.416	Calcium homeostasis [90]
Decarboxylating 3-like	10	33,406,008..33,408,650	0.083	0.010	0.126	0.024	0.670	-0.723	1.416	Maintaining homeostasis during oxidative [91] [92] [93] [94]
Transcription factor MYB3-like	10	33,410,698..33,413,455	0.083	0.010	0.126	0.024	0.670	-0.723	1.416	100 seed weight [42] [43]
Galacturonosyltransferase 9	10	33,424,841..33,427,826	0.083	0.010	0.126	0.024	0.670	-0.723	1.416	Growth, development and stress adaptability [103]
Basic leucine zipper 43-like	10	33,431,425..33,432,396	0.083	0.010	0.126	0.024	0.670	-0.723	1.416	100 seed weight [44]

Table S5. List of candidate genes in QTL for number of seeds per pod on chromosome 9 (7,830,950-8,442,610), differentiation index between subpopulations in QTL and Tajima' D of each subpopulation.

			**Fst**				**Tajima' D**			
**Name**	**Chr.**	**Position**	**pop1 &2**	**pop1 &3**	**pop2 &3**	**all**	**pop1 (KOR)**	**pop2 (JPN)**	**pop3 (CHN)**	**Function**
Cell division protein FtsZ homolog 1, chloroplastic-like	9	7,824,450..7,827,240	0.65	0.004	0.61	0.16	-0.904	0.733	-0.824	Cell wall biosynthesis [148] [149]
TIM22-3-like	9	7,835,374..7,837,849	0.65	0.004	0.61	0.16	-0.904	0.733	-0.824	-
Probable long-chain-alcohol O-fatty-acyltransferase 5	9	7,863,150..7,864,178	0.508	-	0.508	0.122	-0.904	0.733	-0.824	-
Probable long-chain-alcohol O-fatty-acyltransferase 1	9	7,869,513..7,870,821	0.508	-	0.508	0.122	-0.904	0.733	-0.824	-
P21-activated protein kinase-interacting protein 1-like	9	7,875,913..7,888,944	0.508	-	0.508	0.122	-0.904	0.733	-0.824	Signaling [150]
Protein ENHANCED DOWNY MILDEW 2-like	9	7,904,795..7,923,448	0.379	0.006	0.347	0.091	0.896	0.219	0.895	Disease resistance [109]
Uncharacterized protein	9	7,959,709..7,962,123	0.379	0.006	0.347	0.091	0.896	0.219	0.895	-
UDP-glycosyltransferase 79B30-like	9	7,965,725..7,967,086	0.379	0.006	0.347	0.091	0.896	0.219	0.895	-
Putative lipid-transfer protein DIR1	9	7,967,775..7,968,378	0.379	0.006	0.347	0.091	0.896	0.219	0.895	Defense [110] [111]
Sodium/hydrogen exchanger 4	9	7,968,497..7,973,578	0.379	0.006	0.347	0.091	0.896	0.219	0.895	Salt and alkali resistance [112] [113]
Glycerophosphodiester phosphodiesterase GDPDL3-like	9	7,992,707..7,997,898	0.379	0.006	0.347	0.091	0.896	0.219	0.895	Cell wall biosynthesis [162] [152] [153] [154]
Pleckstrin homology domain-containing protein 1-like	9	8,045,432..8,046,229	0.379	0.006	0.347	0.091	0.896	0.219	0.895	-
Glycerophosphodiester phosphodiesterase GDPDL4-like	9	8,052,276..8,053,375	0.379	0.006	0.347	0.091	0.896	0.219	0.895	Cell wall biosynthesis [162] [152] [153] [154]
Glycerophosphodiester phosphodiesterase GDPDL3-like	9	8,072,663..8,080,365	0.379	0.006	0.347	0.091	0.896	0.219	0.895	Cell wall biosynthesis [162] [152] [153] [154]
Pirin-like protein	9	8,129,962..8,133,604	0.288	0.015	0.186	0.09	2.83	-0.198	2.809	-
Beta-(1,2)-xylosyltransferase	9	8,136,366..8,140,171	0.28	0.015	0.18	0.087	2.83	-0.198	2.809	-
40S ribosomal protein S8	9	8,151,956..8,154,564	0.275	0.017	0.169	0.084	2.791	-0.21	2.742	-
LOGL5	9	8,159,729..8,162,635	0.277	0.011	0.189	0.077	2.798	-0.231	2.748	Cytokinin activation [155]
Disease resistance protein RPM1-like	9	8,203,692..8,204,866	0.536	0.004	0.55	0.188	0.893	1.638	-0.644	Disease resistance [114] [115] [116]

Table S6. List of candidate genes in the QTL for number of pods per plant on chromosome 9 (25,969,508-26,273,940), differentiation index between subpopulations in the QTL and Tajima' D of each subpopulation.

			Fst				Tajima' D			
**Name**	**Chr.**	**Position**	**pop1 &2**	**pop1 &3**	**pop2 &3**	**all**	**pop1 (KOR)**	**pop2 (JPN)**	**pop3 (CHN)**	**Function**
Derlin-2.2	9	25,968,859..25,971,853	0.480	0.009	0.409	0.133	-0.218	2.053	-0.620	Maintaining homeostasis during oxidative stress [129] [130]
CDL1-like	9	25,976,891..25,979,083	0.480	0.009	0.409	0.133	-0.218	2.053	-0.620	Growth and development [156]
Pentatricopeptide repeat-containing	9	25,973,195..25,974,746	0.480	0.009	0.409	0.133	-0.218	2.053	-0.620	Embryogenesis and plant development [141] [157]
15.4 kDa class V heat shock protein	9	25,963,805..25,964,900	0.480	0.009	0.409	0.133	-0.218	2.053	-0.620	Heat stress adaptability [132] [158]
Uncharacterized protein	9	25,979,317..25,980,009	0.480	0.009	0.409	0.133	-0.218	2.053	-0.620	-
Beta-glucosidase 47-like	9	26,022,074..26,029,014	0.480	0.009	0.409	0.133	-0.218	2.053	-0.620	Cold resistant [134] [135] [159]
Glucose-6-phosphate translocator 2	9	26,047,539..26,050,354	0.480	0.009	0.409	0.133	-0.218	2.053	-0.620	Environmental signal receptors [136] [137]
Peroxiredoxin-2F, mitochondrial-like	9	26,065,540..26,067,784	0.480	0.009	0.409	0.133	-0.218	2.053	-0.620	Maintaining homeostasis during oxidative stress [139] [160]
Peroxiredoxin-2F, mitochondrial-like	9	26,062,561..26,064,378	0.480	0.009	0.409	0.133	-0.218	2.053	-0.620	Maintaining homeostasis during oxidative stress [139] [160]
Protein trichome birefringence-like 11	9	26,087,449..26,091,648	0.257	0.005	0.217	0.015	-0.218	2.053	-0.620	Growth development and defense [125] [126] [127]
Nuclear TF Y subunit C-3-like	9	26,118,253..26,118,819	0.450	0.397	0.005	0.179	-0.218	2.053	-0.620	Growth and abiotic stress adaptability [161]
Dihydroxy-acid dehydratase	9	26,158,489..26,164,319	0.358	0.291	0.008	0.210	2.702	-0.832	-0.832	Self-resistance [147]
Basic 7S globulin-like	9	26,165,651..26,167,232	0.358	0.291	0.008	0.210	2.702	-0.832	-0.832	Stress response, antibacterial activity [148]
Basic 7S globulin-like	9	26,169,988..26,171,493	0.358	0.291	0.008	0.210	2.702	-0.832	-0.832	Stress response, antibacterial activity [148]
Basic 7S globulin-like	9	26,176,858..26,178,553	0.358	0.291	0.008	0.210	2.702	-0.832	-0.832	Stress response, antibacterial activity [148]
Transcription factor MUTE-like	9	26,199,100..26,200,945	0.358	0.291	0.008	0.210	2.702	-0.832	-0.832	Drought tolerant [149]
Transcription factor MUTE-like	9	26,203,478..26,205,323	0.358	0.291	0.008	0.210	2.702	-0.832	-0.832	Drought tolerant [149]
Neurogenic protein mastermind-like	9	26,238,751..26,241,397	0.380	0.170	0.066	0.188	2.702	-0.832	-0.832	Growth and development [150]
E3 ubiquitin-protein ligase LUL4	9	26,255,522..26,259,528	0.380	0.170	0.066	0.188	2.702	-0.832	-0.832	-
E3 ubiquitin-protein ligase LUL3	9	26,266,303..26,269,400	0.380	0.170	0.066	0.188	2.702	-0.832	-0.832	-
TVP38/TMEM64 family slr0305-like	9	26,283,186..26,290,837	0.273	-	0.273	0.138	0.145	0.041	-0.172	-
Adenylate isopentenyltransferase 5	9	26,325,978..26,327,670	0.273	-	0.273	0.138	0.145	0.041	-0.172	Cytokinin biosynthesis
Serine incorporator 3	9	26,357,666..26,362,827	0.273	-	0.273	0.138	0.145	0.041	-0.172	Virus defense [162]
At5g19020-like	9	26,370,958..26,373,746	0.273	-	0.273	0.138	0.145	0.041	-0.172	Organellar genome expression and biogenesis [141]
